# Supplementary material for: App to support Recovery in Early Intervention Services (ARIES) study: protocol of a feasibility randomised controlled trial of a self-management Smartphone application for psychosis
Source: BMJ Open. 2019 Mar 20;9(3):e025823. doi: 10.1136/bmjopen-2018-025823 (PMC6528051; doi:10.1136/bmjopen-2018-025823)
Supplement: Supplementary data [file bmjopen-2018-025823supp002.pdf]

World Health Organization Trial Registration Data Set

Primary Registry and Trial Identifying Number	ISRCTN10004994
Date of Registration in Primary Registry	25/05/2018
Secondary Identifying Numbers	IRAS ID: 182553<br>REC reference: 15/LO/1453
Source(s) of Monetary or Material Support	The research is funded by the National Institute for Health Research (NIHR) Collaboration for Leadership in Applied Health Research and Care North Thames at Barts Health NHS Trust (NIHR CLAHRC North Thames).
Primary Sponsor	Camden and Islington NHS Foundation Trust
Secondary Sponsor(s)	N/A
Contact for Public Queries	Thomas Steare<br><br>+44 (0)20 7679 8192<br>thomas.steare.15@ucl.ac.uk<br>UCL Division of Psychiatry<br>6th Floor, Maple House<br>149 Tottenham Court Road<br>London<br>W1T 7NF
Contact for Scientific Queries	Prof Sonia Johnson<br><br>+44 (0)20 7679 9453<br>s.johnson@ucl.ac.uk<br>UCL Division of Psychiatry<br>6th Floor, Maple House<br>149 Tottenham Court Road<br>London<br>W1T 7NF
Public Title	ARIES: App to support Recovery in Early Intervention Services
Scientific Title	App to support Recovery In Early intervention Services (the ARIES study): feasibility trial of a supported self-management Smartphone application for psychosis
Countries of Recruitment	England
Health Condition(s) or Problem(s) Studied	First-episode psychosis
Intervention(s)	Self-management Smartphone app (My Journey 3)
Key Inclusion and Exclusion Criteria	Participant inclusion criteria

	1. Currently on the caseload of an Early Intervention Service and in contact with clinicians<br>2. Aged 16 or older<br>3. Have a diagnosis of psychosis<br>4. Own an Android Smartphone.<br><br>Participant exclusion criteria<br><br>1. Lack capacity to provide consent to take part in the study<br>2. Unable to communicate and understand English sufficiently to understand trial procedures and use the app<br>3. In the view of their EIS team, pose such a high risk to others that it would be unsafe to conduct research meetings even on NHS premises.
Study Type	Multi-centre randomised controlled feasibility trial
Date of First Enrolment	09/03/2017
Sample Size	40
Recruitment Status	Recruitment target met – no longer recruiting
Primary Outcome(s)	Relapse as indicated by admission to acute care (inpatient wards, crisis resolution teams, crisis houses and acute day services) during the 12-month follow-up period. Data on admissions to acute care during the trial period is collected from patient records at the 12-month follow-up.
Key Secondary Outcomes	1. Social outcomes are measured using The Social Outcomes Index (Priebe, Watzke, Hansson & Burns, 2008) at the study baseline meeting, at a 4-month follow-up meeting and at a 12-month follow-up meeting<br>2. Mental wellbeing is assessed using The Mental Health Confidence Scale (Carpinello et al., 2000) and The Warwick-Edinburgh Mental Well-Being Scale (NHS Health Scotland, University of Warwick & University of Edinburgh, 2007) at the study baseline meeting, at a 4-month follow-up meeting and at a 12-month follow-up meeting<br>3. Recovery in psychosis is assessed using The Process of Recovery Questionnaire (Neil et al., 2009) at the study baseline meeting, at a 4-month follow-up meeting and at a 12-month follow-up meeting<br>4. Quality of life and satisfaction with treatment is assessed using The DIALOG scale (Priebe et al., 2007) at the study baseline meeting, at a 4-month follow-up meeting and at a 12-month follow-up meeting<br>5. Positive, negative and general psychopathology symptoms are assessed using the PANSS (Kat et al., 1987) at the study

	baseline meeting, at a 4-month follow-up meeting and at a 12-month follow-up meeting<br>6. Participants' engagement with Early Intervention Services during the study period is obtained using the Service Engagement Scale (SES; Tait et al., 2002) completed by participants' clinicians at baseline and at the 12-month follow-up.<br>7. The following patient information is collected from patient records at baseline and one year after entry into the study:<br>7.1. Current diagnosis<br>7.2. Current care cluster<br>7.3. Care plan approach status<br>8. The usability and acceptability of My Journey 3 for service users and clinicians is assessed from semi-structured qualitative interviews conducted at the 4-month follow-up meeting.
Ethics Review	National Research Ethics Service Committee London - Brent, 02/10/2015, ref: 15/LO/1453. Amendment approved 29/07/2017.
Completion date	Study is ongoing
