# Supplementary material for: App to support Recovery in Early Intervention Services (ARIES) study: protocol of a feasibility randomised controlled trial of a self-management Smartphone application for psychosis
Source: BMJ Open. 2019 Mar 20;9(3):e025823. doi: 10.1136/bmjopen-2018-025823 (PMC6528051; doi:10.1136/bmjopen-2018-025823)
Supplement: Author's manuscript [file bmjopen-2018-025823.draft_revisions.pdf]

BMJ Open is committed to open peer review. As part of this commitment we make the peer review history of every article we publish publicly available.

When an article is published we post the peer reviewers' comments and the authors' responses online. We also post the versions of the paper that were used during peer review. These are the versions that the peer review comments apply to.

The versions of the paper that follow are the versions that were submitted during the peer review process. They are not the versions of record or the final published versions. They should not be cited or distributed as the published version of this manuscript.

BMJ Open is an open access journal and the full, final, typeset and author-corrected version of record of the manuscript is available on our site with no access controls, subscription charges or pay-per-view fees (http://bmjopen.bmj.com).

If you have any questions on BMJ Open's open peer review process please email editorial.bmjopen@bmj.com

BMJ Open

The App to support Recovery in Early Intervention Services (ARIES) Study: Protocol of a feasibility randomised controlled trial of a self-management Smartphone application for psychosis

Journal:	*BMJ Open*
Manuscript ID	bmjopen-2018-025823
Article Type:	Protocol
Date Submitted by the Author:	08-Aug-2018
Complete List of Authors:	Steare, Thomas; University College London, Division of Psychiatry; Camden and Islington NHS Foundation Trust,<br>O'Hanlon, Puffin; University College London, Division of Psychiatry<br>Eskinazi, Michelle; University College London, Division of Psychiatry<br>Osborn, David; UCL, Faculty of Brain Sciences; Camden and Islington NHS Foundation Trust<br>Lloyd-Evans, Brynmor ; UCL, Division of Psychiatry; Camden and Islington NHS Foundation Trust<br>Jones, Rebecca; UCL, Division of Psychiatry<br>Rostill, Helen; Surrey and Borders Partnership NHS Foundation Trust<br>Amani, Sarah; NHS England, Early Intervention in Psychosis Programme (South of England)<br>Johnson, Sonia; UCL, Division of Psychiatry ; Camden and Islington NHS Foundation Trust
Keywords:	Schizophrenia & psychotic disorders < PSYCHIATRY, MENTAL HEALTH, PSYCHIATRY

SCHOLARONE™
Manuscripts

TITLE PAGE

Title: The App to support Recovery in Early Intervention Services (ARIES) Study: Protocol of a feasibility randomised controlled trial of a self-management Smartphone application for psychosis

Authors:

Thomas Steare

Division of Psychiatry, University College London, London, UK

R&D Department, Camden and Islington NHS Foundation Trust, London, UK

Puffin O'Hanlon

Division of Psychiatry, University College London, London, UK

Michelle Eskinazi

Division of Psychiatry, University College London, London, UK

David Osborn

Division of Psychiatry, University College London, London, UK

R&D Department, Camden and Islington NHS Foundation Trust, London, UK

Brynmor Lloyd-Evans

Division of Psychiatry, University College London, London, UK

R&D Department, Camden and Islington NHS Foundation Trust, London, UK

Rebecca Jones

Division of Psychiatry, University College London, London, UK

Helen Rostill

Surrey and Borders Partnership NHS Foundation Trust, Leatherhead, Surrey, UK

For peer review only

Sarah Amani

Senior Programme Manager, EIP Programme (South of England), NHS England, Oxford, Oxfordshire, UK

Sonia Johnson (Corresponding author)

Address: UCL Division of Psychiatry, 6th Floor, Wing B, Maple House, 149 Tottenham Court Road, London W1T 7NF

Division of Psychiatry, University College London, London, UK

R&D Department, Camden and Islington NHS Foundation Trust, London, UK

s.johnson@ucl.ac.uk

02076799453

Trial Sponsor: Camden & Islington NHS Foundation Trust. sponsor.noclor@nhs.net

Study start date (first participant recruited): 09 March 2017

Study end date: 30 November 2018

Keywords: Psychosis, Self-management, Smartphone, Feasibility study, Randomised controlled trial

Word count: 3,999

For peer review only

ABSTRACT

Introduction: Mental health interventions delivered through digital technology have potential applications in promoting recovery and improving outcomes among people in the early stages of psychosis. Self-management approaches are recommended for the treatment of psychosis and could be delivered via applications (apps) installed on Smartphones to provide low-cost accessible support. We describe the protocol for a feasibility trial investigating a self-management Smartphone app intervention for adults using Early Intervention in Psychosis (EIP) services.

Methods and analysis: In this feasibility randomised controlled trial, 40 participants will be recruited from EIP services in London and Surrey. Twenty participants will be randomised to receive a supported self-management Smartphone app (My Journey 3) plus Treatment As Usual (TAU), while the other 20 participants will receive TAU only. The primary objective of this study is to evaluate the feasibility of conducting a full-scale trial of this intervention in EIP services. Participant data will be collected at baseline and at two follow-up assessments conducted four months and 12 months post-baseline. Outcome measures will include admission to acute care, mental health and wellbeing, recovery, quality of life and psychopathology. Semi-structured interviews with participants and EIP service clinicians will additionally explore experiences of using My Journey 3 and participating in the trial and suggestions for improving the intervention.

Ethics and dissemination: The ARIES study has been reviewed and approved by the National Research Ethics Service Committee London – Brent (Research Ethics Committee reference: 15/LO/1453). The findings of this study will be disseminated through peer-reviewed scientific journals and conferences, magazines and web publications.

Trial registration number: ISRCTN10004994

For peer review only

ARTICLE SUMMARY

Strengths and limitations of this study

- This study tests the feasibility of a randomised controlled trial for a Smartphone app which is already well developed: there has been substantial stakeholder input to development at all stages, an initial period of testing in clinical settings, and refinement of the current version through lab and field testing.
- There is a strong rationale and substantial evidence for supported self-management in psychosis, but implementation is not currently widespread: the tool tested has potential to deliver more extensive implementation.
- The trial will only recruit Android Smartphone users as My Journey 3 is not as yet compatible with iOS or other Smartphone operating systems.
- Blinding has not been feasible in this trial.

For peer review only

INTRODUCTION

Psychosis is associated with significant costs at individual-, family- and society-level.[1-3] The implementation of Early Intervention in Psychosis (EIP) services across the United Kingdom has been associated with improved outcomes,[4,5] however substantial challenges remain. Long-term follow-up studies have shown that relapse rates in the early course of psychosis remain high.[6] Furthermore functional recovery is often not attained following treatment.[7]

Thus there is a need to increase and sustain benefits from EIP services. An approach with demonstrated efficacy in improving functional recovery and reducing relapse in psychosis is supported self-management.[8] Self-management has been designed to empower people as active agents in their own recovery by enabling them to develop skills such as recognising and monitoring symptoms and early warning signs of relapse, identifying and avoiding stressors, and using effective coping strategies.[9] Self-management is associated with significant benefits including reduced distress, improved medication adherence and reduced number of hospitalizations.[10-12] Relapse prevention work has long been proposed as a key element in EIP,[13] and establishing and working towards recovery goals is widely advocated in EIP services.[14] However, well-evaluated tools and methods are currently not available to support widespread implementation of self-management approaches in EIP services.

Over recent years there has been growing interest in the development of innovative technologies in health-care due to their potential to improve accessibility, efficacy, quality and cost-effectiveness of treatment.[15] Due to rapid advancements in mobile phone technology, it is possible to deliver clinical interventions via applications (apps) installed on Smartphones (mobile phones with computational capacities). Smartphones are widely

For peer review only

available in the UK with over 75% of adults owning one.[16] They are often carried on the person with high accessibility to the internet and are therefore a suitable device to provide time-unlimited interventions in almost any location.[17]

Recent evidence suggests that people with psychosis are adopting digital technology in a similar way to the general population,[18-20] and that they are interested in using mental health interventions delivered via Smartphones.[21,22] A systematic review has found that interventions delivered through apps or text messaging on Smartphones are acceptable and feasible for people with psychosis and may support recovery.[23] Smartphone apps based on self-management principles have shown promise in a wide range of long-term health conditions,[24] and are a potential way to deliver accessible low-cost support to adults with psychosis.

Although emerging research suggests that Smartphone apps hold promise in delivering effective interventions to adults with psychosis, the evidence base is under-developed in comparison to more common mental disorders.[25] To date only one randomised controlled trial (RCT) of a psychosocial Smartphone app used in EIP services has been published.[26] In this proof-of-concept trial, 36 adults accessing EIP services were randomised to use a cognitive-behavioural therapy informed Smartphone app (Actissist) that aims to encourage active self-management, or a symptom-monitoring app which was classed as the control condition. Actissist was found to be acceptable, feasible and safe for adults accessing EIP services. Although not powered to find an effect, the study suggests that Actissist may confer benefits to users' outcomes over and above a passive symptom-monitoring app. Smartphone apps that promote self-management therefore have the potential to improve outcomes in first-episode psychosis and reduce healthcare costs and need investigating further in RCTs.

For peer review only

Aims and objectives

The aim of the ARIES study is to examine the feasibility of conducting a full-scale trial of a clinician supported self-management Smartphone app for adults accessing EIP services. The feasibility trial will aim:

1. To identify whether a self-management Smartphone app is acceptable to use and feasible to support in EIP services in the context of a research study, and to identify any necessary modifications to the intervention content and design, or to its delivery in EIP services.
2. To test the feasibility and acceptability of trial procedures for a definitive trial.
3. To test procedures for evaluating intervention engagement and participant outcomes.
4. To assess recruitment and retention rates to inform planning of a definitive trial.

METHODS AND ANALYSIS

Design

The study is a feasibility RCT comparing a clinician supported self-management Smartphone app (My Journey 3), in addition to TAU, to a control group receiving TAU only. The design as described here adheres to the Standard Protocol Items: Recommendations for Interventional Trials (SPIRIT).[27] A copy of the SPIRIT checklist is provided as Additional file 1. Relevant items from the World Health Organization Trial Registration Data Set are detailed in Additional file 2.

Setting

The trial will take place in six EIP services across three NHS Foundation Trusts in England; Camden and Islington, East London, and Surrey and Borders Partnership. All

For peer review only

participating EIP services provide care coordination to service users, access to a psychiatrist and psychiatric medication and psychosocial interventions, aiming to conform to the current UK model for EIP services.[28] None of the participating EIP services offer Smartphone apps or any other digital interventions as part of routine care, nor are structured self-management tools consistently available in these teams. A list of participating sites is available from the authors.

Participants

Forty service users will be recruited from the participating EIP services. The proposed sample size has been chosen to be sufficient to establish the acceptability of My Journey 3 and the feasibility of trial procedures.[29]

Eligibility criteria

Inclusion criteria

- Aged 16 or older
- Have experienced at least one episode of psychosis
- Currently on the caseload of an EIP service and in contact with clinicians
- User of a Smartphone with an Android operating system.

Exclusion criteria

- Lack of capacity to provide informed consent to participate in the trial
- Inability to communicate and understand English sufficiently to understand trial procedures and use My Journey 3
- In the view of their EIP service, poses such a high risk to others that it would be unsafe to conduct research meetings even on NHS premises.

For peer review only

Criteria are deliberately broad in order to reach conclusions generalisable to a wide range of EIP service users. Due to limited resources My Journey 3 has been developed for one Smartphone operating system (Android) at this stage of testing. Nearly half of Smartphones used in the UK utilise Android operating systems.[30] There is also emerging evidence that Android may be the Smartphone operating system most often used by adults with severe mental illness.[21] Resources are not currently available to deliver My Journey 3 in languages other than English.

Recruitment

An overview of the recruitment procedure is displayed in the flow diagram (figure 1). Clinicians will identify EIP service users who appear potentially eligible for the trial. They will then make initial contact, explain the trial and ask if the potential participant is willing to be contacted by a researcher. If a potential participant is eligible and interested in taking part, a researcher will contact them to further explain the trial and to arrange a face-to-face meeting. At this meeting the researcher will provide the trial information sheet, confirm eligibility, invite any questions about participating and assess the service user's capacity to provide informed consent. Written consent to participate will be obtained using a consent form (Additional file 3) prior to the baseline assessment.

Randomisation

After completion of the baseline assessment participants will be randomly allocated in a 1:1 ratio to an intervention group where they will have access to My Journey 3 during the trial (n=20) or to a control group (n=20). Randomisation will be conducted by an independent statistician. The allocation sequence will be concealed from the researcher, who will be blind when recruiting participants and conducting baseline assessments. Following randomisation participants will be informed of their allocation by the researcher. Participants cannot be blinded to their group allocation given the nature of the trial intervention and control group.

For peer review only

As a single researcher will carry out most of the data collection, it is not practical for the group allocation of participants to be concealed from the research team.

The intervention

Development

My Journey 3 has been developed through a series of iterations. The first version of My Journey was designed by Surrey and Borders Partnership NHS Foundation Trust (led by Sarah Amani), after local EIP service users consulted about their care suggested there would be significant benefits from a Smartphone app that could be used for appointment and medication reminders, to track their mood and to share their recovery progress with EIP staff or carers. In 2011 a project group consisting of EIP service users, NHS clinicians, a pharmacist and a NHS manager was formed to drive the development of the My Journey app. The following year a prototype of My Journey was tested by 20 EIP service users to further inform development of the app. Since April 2013 this version of My Journey has been available for download from Google Play (the official app store for Android Smartphones).

The original version of My Journey contains generic advice on what to do if certain clinical difficulties arise, but does not allow personalised relapse prevention planning or recovery goal tracking. User feedback suggested that this was a limitation. In the current study we have collaborated with digital health experts, EIP service clinicians and adults with lived experience of psychosis to adapt existing paper-and-pen self-management intervention components – in routine use in NHS services - to be suitable for delivery in an app format [31,32]. In creating the product specification of My Journey 3 we have incorporated these self-management components with functions from the original My Journey.

For peer review only

Following the initial development of My Journey 3, it has been refined in response to two phases of preliminary testing. In the first phase six volunteer EIP services users participated in usability lab tests with My Journey 3 on their own Android Smartphone. Usability testing featured "think aloud" tests where participants completed set tasks using My Journey 3 while providing a continuous commentary on their thoughts. This method was used to highlight design and usability issues and users' immediate reactions to the app. Individual interviews were also conducted to explore participants' perceptions of the ease of use of My Journey 3, concerns they might have and suggestions for improvements. My Journey 3 was modified to reflect the findings at this stage.

In the second phase a further six volunteer service users trialled My Journey 3 on their own Android Smartphone during a one-month field study, with EIP service clinicians asked to support their clients' use of the app during routine appointments. Following the field study, individual interviews were conducted with service user participants to explore how they used My Journey 3 and how it could be improved. Further interviews with EIP service clinicians explored their experience of supporting their client with My Journey 3. Based on these findings My Journey 3 was updated again prior to the feasibility trial.

Intervention outline

My Journey 3 is a Smartphone app that has been designed to be used alongside EIP service care and with the support of clinicians. My Journey 3 aims to develop and support users' illness self-management skills to help facilitate recovery from first-episode psychosis. The My Journey 3 home screen can be seen in figure 2.

Three main components of My Journey 3 have been directly taken from the original app and updated. Information regarding psychosis, mental health and mental health services is provided in My Journey 3 through links to relevant NHS and voluntary sector websites and

For peer review only

through videos of personal recovery stories. To facilitate symptom recognition and monitoring My Journey 3 also features a self-monitoring tool and symptom tracker where users can monitor 14 different symptoms and 3 lifestyle behaviours. Advice to help manage symptoms is provided after completing the symptom tracker, and a graphical summary of symptom severity over time is displayed. Users also have access to a pill tracker where they can log whether they have taken their psychiatric medication. The pill tracker features a daily alert at a pre-set time to remind users to input if they have taken their medication.

In designing My Journey 3 we have also drawn on evidenced-based self-management interventions to add structured intervention components focused on recovery planning and relapse prevention.[31,32] These additions allow for users to interactively:

- Identify strategies and coping resources that they find useful in maintaining wellbeing
- Set and track progress towards personal recovery goals
- Identify personal early warning signs of relapse and strategies and coping mechanisms to put in place should they experience these
- Create a “relapse plan”, an action plan to follow in times of crisis in order to avoid or attenuate relapse

My Journey 3 has been designed to be used by EIP service users in collaboration with clinicians. Clinicians can input relevant information to sections such as the relapse prevention plans and, with training, can provide assistance with the app. It is also suitable for independent use: the developers’ aspiration is for the app to be initially used in collaboration with clinicians, but for it then to support users’ self-management following discharge from EIP services.

For peer review only

My Journey 3 features weekly discrete notifications that appear on the users' Smartphone interface to encourage engagement with the app. Users also have the opportunity to set activity reminders that notify the user to engage in coping strategies and take part in pre-identified activities to promote wellbeing.

My Journey 3 also features a sharing functionality for users who wish to share their data with clinicians, family members, friends or other trusted third parties. This uses built-in sharing functionality of the user's Smartphone, such as e-mail. Participants will have control of who they choose to share their data with at all times.

Delivery of the intervention

Each participant in the intervention group will take part in an individual training session with their supporting EIP service clinician and a researcher within six weeks of the initial consent meeting. During each training session participants will download My Journey 3 on to their Smartphone. The researcher will then give a demonstration of the app and its main functions. Participants will have the opportunity to practice using My Journey 3 and to ask questions. To facilitate use during the trial participants will be asked to input appropriate information in to the main functions of My Journey 3. The researcher will encourage participants and supporting EIP service clinicians to discuss recovery goals and relapse prevention plans in following routine appointments and then to input them into My Journey 3. Participants will have access to My Journey 3 from the training session till the 12-month time-point.

Control group

Participants in the control group (n=20) will receive TAU that will be unaffected by their participation in this trial.

Treatment As Usual

For peer review only

TAU for service users attending EIP services typically involves regular meeting with a care co-ordinator, support from multi-disciplinary clinicians and access to a psychiatrist, psychiatric medication, and a range of psychological interventions.

Data collection

Baseline and follow-up assessments

The participant timeline is summarised in table 1. All participants will be asked to complete self-report questionnaires during a structured assessment with a researcher at three time points; baseline (prior to randomisation), 4-months post baseline and 12-months post baseline. Prior to the completion of each assessment participants will be provided with a trial information sheet. Their capacity to give informed consent will be assessed and their consent documented in writing. Participants will receive 20 pounds as a token of thanks for completing each assessment.

For peer review only

Table 1. Timeline of participant enrolment, interventions, assessments and patient records data collection

Time-point	Enrolment	Baseline	Allocation	4-month follow-up	12-month follow-up
**Enrolment:**					
Eligibility screen	X				
Informed consent		X			
Randomisation			X		
**Intervention:**					
My Journey 3 (intervention group)			←	—	→
TAU (all participants)		←	—	—	→
**Assessments:**					
PANSS		X		X	X
Sociodemographic information		X		X	X
Clinical service use		X		X	X
Social Outcomes Index		X		X	X
Mental Health Confidence Scale		X		X	X
Questionnaire about the Process of Recovery		X		X	X
WEMWBS		X		X	X
The DIALOG Scale		X		X	X
Service Engagement Scale (completed by EIP service clinicians)		X			X
Qualitative interviews (with participants in the intervention group and supporting clinicians)				X	
**Patient records data (from previous 12 months to time point):**					
Number of admissions to acute mental health services		X			X
Number of compulsory admissions to acute mental health services		X			X
Total number of days in acute care		X			X
Number of kept appointments with community mental health services		X			X
Number of missed appointments with community mental health services		X			X
Primary ICD-10 diagnosis		X			X

Most recent care cluster		X			X
Care Programme Approach status		X			X

WEMWBS Warwick-Edinburgh Mental Well-being Scale, *PANSS* Positive and Negative Syndrome Scale

Qualitative interviews

During the completion of the four-month follow-up assessments, participants in the intervention group will be invited to complete an audio-recorded interview with a researcher. The interviews will follow a topic guide and will explore participants' experience of using My Journey 3, including:

- The usability and acceptability of My Journey 3
- Positives and negative aspects of My Journey 3
- Impact of My Journey 3 on their life
- Facilitators and barriers to using My Journey 3
- Views on the training session
- Views on the support they received from their clinician in using My Journey 3

EIP service clinicians who have been supporting participants with My Journey 3 will also be asked to complete an audio-recorded interview close to the time the participant completes the four-month follow-up assessment. Written consent to take part will be confirmed beforehand, with an information sheet provided. Clinicians will be asked to provide demographic data prior to undertaking the interview. The interview will follow a topic guide and will focus on:

- Positives and negative aspects of My Journey 3
- The experience of supporting clients with My Journey 3

For peer review only

- Facilitators and barriers to providing support and to the incorporation of My Journey 3 in clinical management
- Views on the training session

Data from patient records

Once the recruitment target of the study has been met, the researcher will contact the appropriate administrators or informatics team within each NHS trust to arrange the collection of participant data from patient records. The researcher will also arrange for data to be collected from patient records one year later. Data from patient records will include participants' use of mental health services during the 12-months prior to entering the study, and during the 12-month trial period.

My Journey 3 usage data

Data regarding My Journey 3 use will be collected throughout the trial period for all participants in the intervention group. My Journey 3 will automatically upload encrypted usage data to a secure trial server when the user has internet access on their Smartphone. The data collected will be a record of each time the user opens My Journey 3, whether this was in response to a prompt, and which components they use. Usage data will be used to assess the acceptability of My Journey 3 and user engagement with it.

Outcomes

For peer review only

The feasibility trial is not powered to test hypotheses, but assess the feasibility of participant outcome measures for use in a future RCT. Participant outcome data will be collected from assessments with a researcher and from patient records.

The proposed primary outcome for a future fully-powered RCT: Whether participants experience a relapse over one year following study entry (indicated by admission to an acute care setting, including acute inpatient wards, crisis resolution teams, crisis houses and acute day care services).

The following are measured as potential secondary outcomes for a future fully-powered RCT;

Service use measures over 1 year of follow-up

1. Engagement with EIP services as measured by the Service Engagement Scale (SES),[33] a 14-item questionnaire that measures engagement for four different dimensions; availability, collaboration, help-seeking and treatment adherence. The SES will be completed by EIP service clinicians, such as participants' care co-ordinators, at baseline and at the 12-month time-point.

Measures at baseline, 4-month and 12-month follow-up assessments

1. Psychotic symptoms and general psychopathology, measured by the Positive and Negative Syndrome Scale (PANSS), a 30-item scale that yields three separate scores on positive symptoms, negative symptoms and general psychopathology.[34] To inform the PANSS a trained researcher will conduct clinical interviews with participants at each assessment.
2. Social outcomes, rated by the Social Outcomes Index (SIX),[35] a 6-item index of social outcomes and circumstance.

For peer review only

3. Mental health-related self-efficacy, measured by the Mental Health Confidence Scale (MHCS),[36] a 16-item self-report scale of service users' confidence in their ability to cope with stressful or difficult events.
4. Self-rated recovery measured by the Questionnaire about the Process of Recovery (QPR),[37] a 22-item measure, yielding a total score and subscale scores for interpersonal and interpersonal recovery factors.
5. Mental wellbeing, rated by total score on The Warwick-Edinburgh Mental Well-Being Scale (WEMWBS),[38] a 14-item self-report scale of mental wellbeing.
6. Subjective quality of life and satisfaction with treatment measured by The DIALOG scale,[39] an 11-item self-report scale.

Further measures that will be collected to characterise the sample include the following:

a. Sociodemographic characteristics including age, gender and ethnicity. We will also collect data regarding accommodation and living situation, employment status, educational attainment and Smartphone use.
b. Clinical diagnosis as recorded in patient records using the ICD-10 (Internal Classification of Diseases: 10th Revision) classification.
c. Service use during the previous 12 months measured at baseline and 12-months post study entry. Participants' service use will be collected from patient records and will include data on inpatient admissions and compulsory admissions, the number of kept and missed appointments with mental health services, most recent care cluster and care programme approach status.

Data analysis

Quantitative analysis

For peer review only

We will report rates of recruitment and retention in the trial, and, for the intervention group, the level of usage of My Journey 3 during the trail. The demographic and clinical characteristics of participants at baseline will be summarised separately for each study group using descriptive statistics.

To pilot the methods of analysis for a full-scale trial we will use an intention-to-treat approach using data from all randomised participants. The effect of My Journey 3 on relapse during the 12 months of the trial will be estimated using logistic regression. The effect of the intervention on continuous outcome measures (SIX, QPR, WEMWBS, DIALOG scale, PANSS and SES) will be estimated using linear regression adjusting for the baseline measure of the outcome in question. Results will be summarised using effect estimates and 95% confidence intervals only. No interim analyses are planned.

Qualitative analysis

Interview data with participants in the intervention group and supporting EIP service clinicians will be analysed using thematic analysis.[40] Analyses will be conducted collaboratively by a group of researchers. The analysis will focus on participants' and EIP service clinicians' experience of My Journey 3, aspects that could be improved and the acceptability of My Journey 3.

Patient and Public Involvement

Independent advice will be sought from a trial steering group. Members will include researchers with expertise in developing and testing digital health interventions, EIP service clinicians, people with lived experience of first-episode psychosis and carers. Steering group members with lived experience of psychosis were consulted on the first paper prototypes of My Journey 3 and on the research protocol. The version of My Journey 3 tested in the current feasibility trial has been developed based on EIP service users' feedback from the usability

For peer review only

tests and field study. Participants will be offered a summary of the research findings at the end of the study.

Data monitoring and management

A secure password-protected trial database will be developed and managed to store all quantitative data using SPSS software V.23, and will feature non-identifiable trial IDs only. Data entry of participants' questionnaires will be primarily undertaken by the trial researcher, with a random sample checked by other research team members. Anonymised electronic interview transcripts will be checked for accuracy and stored using NVivo for Windows (QSR International Pty Ltd V.11, 2016). After the trial, all data will be archived securely at University College London.

Due to the small sample size of the study a data monitoring committee is not planned, but the steering group will advise if one is later needed.

ETHICS AND DISSEMINATION

Ethical approval

Ethical approval has been obtained from the London Brent National Research Ethics Service Committee (Research Ethics Committee reference: 15/LO/1453), which has approved all amendments to protocol. Future protocol modifications will be submitted for approval to the research ethics committee and communicated to the study sponsor, site principle investigators, participating NHS trusts and participants. The current protocol in use is V.9, 29 July 2017.

Confidentiality

Participant data will be accessed by the research team only. Consent forms and data collection forms will be securely stored in locked cabinets at University College London.

For peer review only

Data collection forms will not feature participants' names but a unique trial ID that could not be linked to participants by anyone outside the research team. Consent forms that identify participants will be kept separately from data collection forms.

Password-protected electronic data will be stored on the secure IT network at University College London. App usage data collected whilst using My Journey 3 will be anonymised and encrypted and will not contain personal user information such as text input or responses to self-rated questions.

For peer review only

Serious adverse events

Serious adverse events such as hospital admissions and death that have been reported to the trial team will be reviewed by the Chief Investigator. Identified adverse events assessed as trial-related will be reported to the trial sponsor.

Dissemination

Results will be disseminated through scientific publications, and to a wider audience via magazines and web publications.

Acknowledgements: The ARIES research team are grateful to their software collaborators MyOxygen for their technical development and hosting of My Journey 3 and to Ali Mousa for his valuable contribution to the development of the original My Journey app. We are grateful to Max Birchwood for his permission to incorporate 'Back in the Saddle' in to My Journey 3. We are grateful to Rachel Perkins for her permission to adapt the Personal Recovery Plan resource and incorporate in to My Journey 3.

Author Contributions: The trial design was developed by SJ, DO, BLE and PO. SA, HR, PO and ME have led on the development of the intervention. RJ has advised on the statistical analysis. SJ is the Chief Investigator, based at University College London, DO the co-Chief Investigator, and TS the project manager. All authors have contributed and approved this manuscript.

Funding: The research is funded by the National Institute for Health Research (NIHR) Collaboration for Leadership in Applied Health Research and Care North Thames at Barts Health NHS Trust (NIHR CLAHRC North Thames). The views expressed in this article are those of the authors and not necessarily those of the NHS, the NIHR or the Department of Health and Social Care.

For peer review only

Data statement: The datasets generated during and/or analysed during the current study will be made available two years after the trial end.

Competing interests: None declared.

Legends:

Figure 1. SPIRIT flow diagram outlining the phases of the ARIES feasibility trial.

Figure 2. The My Journey 3 home screen, which is seen by the user when accessing the app on their Smartphone.

REFERENCES

[1] Addington J, Young J, Addington D. Social outcome in early psychosis. Psychol Med. 2003;33:1119-24.

[2] Addington J, Coldham EL, Jones B, Ko T, Addington D. The first episode of psychosis: the experience of relatives. Acta Psychiat Scand. 2003;108:285-9.

[3] The Schizophrenia Commission. The abandoned illness: a report from the Schizophrenia Commission. London: Rethink Mental Illness. 2012.

[4] Bird V, Premkumar P, Kendall T, Whittington C, Mitchell J, Kuipers E. Early intervention services, cognitive–behavioural therapy and family intervention in early psychosis: systematic review. Brit J Psychiat. 2010;197:350-6.

[5] Tsiachristas A, Thomas T, Leal J, et al. Economic impact of early intervention in psychosis services: results from a longitudinal retrospective controlled study in England. BMJ Open 2016;6:e012611. doi: 10.1136/bmjopen-2016-012611

For peer review only

[6] Alvarez-Jiménez M, Parker AG, Hetrick SE, McGorry PD, Gleeson JF. Preventing the second episode: a systematic review and meta-analysis of psychosocial and pharmacological trials in first-episode psychosis. Schizophrenia Bull. 2009;37:619-30.

[7] Menezes NM, Arenovich T, Zipursky RB. A systematic review of longitudinal outcome studies of first-episode psychosis. Psychol Med. 2006;36:1349-62.

[8] Mueser KT, Deavers F, Penn DL, Cassisi JE. Psychosocial treatments for schizophrenia. Annu Rev Clin Psycho. 2013;9:465-97.

[9] Mueser KT, Meyer PS, Penn DL, Clancy R, Clancy DM, Salyers MP. The Illness Management and Recovery program: rationale, development, and preliminary findings. Schizophrenia Bull. 2006;32:S32-43.

[10] Mueser KT, Corrigan PW, Hilton DW et al. Illness management and recovery: A review of the research. Psychiat Serv. 2002;53:1272-84.

[11] Cook JA, Copeland ME, Jonikas JA, et al. Results of a randomized controlled trial of mental illness self-management using Wellness Recovery Action Planning. Schizophrenia Bull. 2011;38:881-91.

[12] Färdig R, Lewander T, Melin L, Folke F, Fredriksson A. A randomized controlled trial of the illness management and recovery program for persons with schizophrenia. Psychiat Serv. 2011;62:606-12.

[13] Birchwood M, Spencer E, McGovern D. Schizophrenia: early warning signs. Advances in Psychiatric Treatment. 2000;6:93-101.

[14] Slade M. Personal Recovery and Mental Illness: A Guide for Mental Health Professionals. Cambridge: Cambridge University Press; 2009. (Values-Based Practice).

For peer review only

[15] Department of Health. Closing the Gap: Priorities for Essential Change in Mental Health. 2014, https://www.gov.uk/government/uploads/system/uploads/attachment_data/file/281250/Closing_the_gap_V2_-_17_Feb_2014.pdf (accessed August 2018)

[16] Ofcom. The Communications Market Report. London, UK: Ofcom; 2018

[17] Proudfoot J. The future is in our hands: the role of mobile phones in the prevention and management of mental disorders. Aust NZ J Psychiat. 2013;47:111-3.

[18] Firth J, Cotter J, Torous J, Bucci S, Firth JA, Yung AR. Mobile phone ownership and endorsement of “mHealth” among people with psychosis: a meta-analysis of cross-sectional studies. Schizophrenia Bull. 2015;42:448-55.

[19] Robotham D, Satkunanathan S, Doughty L, Wykes T. Do We Still Have a Digital Divide in Mental Health? A Five-Year Survey Follow-up. J Med Internet Res. 2016;18:e309.

[20] Naslund JA, Aschbrenner KA, Bartels SJ. How people with serious mental illness use smartphones, mobile apps, and social media. Psychiatr Rehabil J. 2016;39:364-367.

[21] Ben-Zeev D, Kaiser SM, Brenner CJ, Begale M, Duffecy J, Mohr DC. Development and usability testing of FOCUS: A smartphone system for self-management of schizophrenia. Psychiatr Rehabil J. 2013;36:289-296.

[22] Aref-Adib G, O’Hanlon P, Fullarton K, et al. A qualitative study of online mental health information seeking behaviour by those with psychosis. BMC Psychiatry. 2016;16:232.

[23] Bonet L, Izquierdo C, Escartí MJ, et al. Use of mobile technologies in patients with psychosis: A systematic review. Rev Psiquiatr Salud Ment. 2017;10:168-78.

For peer review only

[24] Whitehead L, Seaton P. The effectiveness of self-management mobile phone and tablet apps in long-term condition management: a systematic review. J Med Internet Res. 2016;18:e97.

[25] O'Hanlon P, Aref-Adib G, Fonseca A, et al. Tomorrow's world: current developments in the therapeutic use of technology for psychosis. BJPsych Advances. 2016;22:301-10.

[26] Bucci S, Barrowclough C, Ainsworth J, et al. Actissist: Proof-of-Concept Trial of a Theory-Driven Digital Intervention for Psychosis. Schizophrenia Bull. 2018; sby032.

[27] Chan A-W, Tetzlaff JM, Gøtzsche PC, et al. SPIRIT 2013 explanation and elaboration: guidance for protocols of clinical trials. BMJ. 2013;346:e7586

[28] National Institute for Health and Care Excellence. Implementing the Early Intervention in Psychosis Access and Waiting Time Standard: Guidance. London: NHS England Publications. 2016.

[29] Julious, SA. Sample size of 12 per group rule of thumb for a pilot study. Pharm Stat. 2005;287-91.

[30] Ofcom. The Communications Market Report. London, UK: Ofcom; 2017

[31] Plaistow J, Birchwood M. Back in the saddle: a guide to relapse prevention. In: Birchwood M, Fowler D, Jackson C, editors. Early Intervention in Psychosis: A Guide to Concepts, Evidence and Interventions. Chichester: McGraw-Hill; 1996. p. 239-244.

[32] Perkins R, Rinaldi M. Taking back control: a guide to planning your own recovery. South West London: St George's Mental Health NHS Trust, 2007.

[33] Tait L, Birchwood M, Trower P. A new scale (SES) to measure engagement with community mental health services. J Ment Health. 2002;11:191-8.

For peer review only

[34] Kay SR, Fiszbein A, Opfer LA. The positive and negative syndrome scale (PANSS) for schizophrenia. Schizophrenia Bull. 1987;13:261-76.

[35] Priebe S, Watzke S, Hansson L, et al. Objective social outcomes index (SIX): a method to summarise objective indicators of social outcomes in mental health care. Acta Psychiat Scand. 2008;118:57-63.

[36] Carpinello SE, Knight EL, Markowitz FE, et al. The development of the Mental Health Confidence Scale: A measure of self-efficacy in individuals diagnosed with mental disorders. Psychiatr Rehabil J. 2000;23:236-243.

[37] Neil ST, Kilbride M, Pitt L, et al. The questionnaire about the process of recovery (QPR): a measurement tool developed in collaboration with service users. Psychosis. 2009;1:145-55.

[38] Tennant R, Hiller L, Fishwick R, et al. The Warwick-Edinburgh mental well-being scale (WEMWBS): development and UK validation. Health Qual Life Out. 2007;5:63.

[39] Priebe S, McCabe RO, Bullenkamp J, et al. Structured patient–clinician communication and 1-year outcome in community mental healthcare. Brit J Psychiat. 2007;191:420-6.

[40] Braun V, Clarke V. Using thematic analysis in psychology. Qual Res Psychol. 2006;3:77-101.

For peer review only

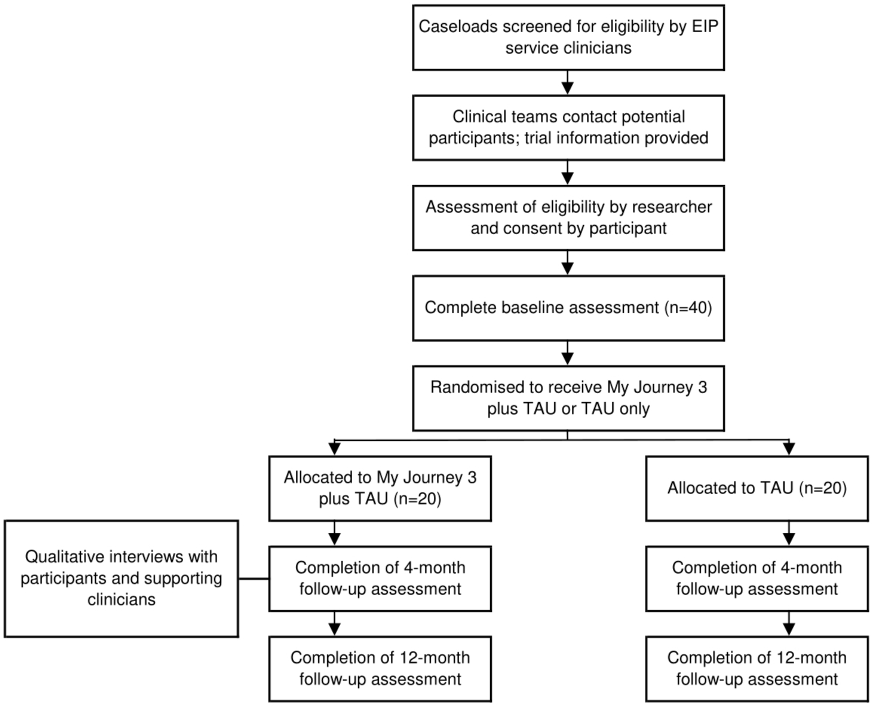

SPIRIT flow diagram outlining the phases of the ARIES feasibility trial.

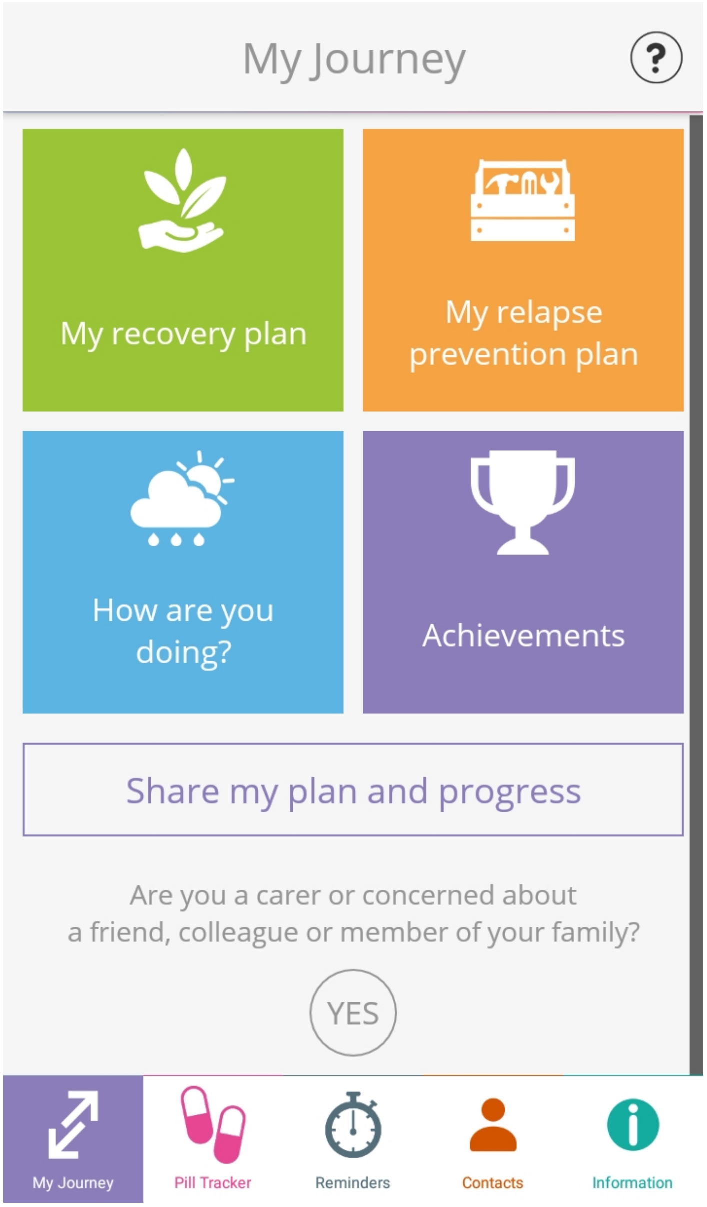

The My Journey 3 home screen, which is seen by the user when accessing the app on their Smartphone.

SPIRIT

STANDARD PROTOCOL ITEMS: RECOMMENDATIONS FOR INTERVENTIONAL TRIALS

SPIRIT 2013 Checklist: Recommended items to address in a clinical trial protocol and related documents*

**Section/item**	**Item No**	**Description**	**Addressed on page number**
**Administrative information**			*Sections headings given until page numbers finalised*
Title	1	Descriptive title identifying the study design, population, interventions, and, if applicable, trial acronym	Title page
Trial registration	2a	Trial identifier and registry name. If not yet registered, name of intended registry	Abstract page
	2b	All items from the World Health Organization Trial Registration Data Set	Additional file 2
Protocol version	3	Date and version identifier	Ethics and dissemination - Ethical approval
Funding	4	Sources and types of financial, material, and other support	Declarations - funding
Roles and responsibilities	5a	Names, affiliations, and roles of protocol contributors	Title page/ Declarations - authors' contributions
	5b	Name and contact information for the trial sponsor	Title page

For peer review only

For peer review only - http://bmjopen.bmj.com/site/about/guidelines.xhtml

	5c	Role of study sponsor and funders, if any, in study design; collection, management, analysis, and interpretation of data; writing of the report; and the decision to submit the report for publication, including whether they will have ultimate authority over any of these activities	Declarations - funding
	5d	Composition, roles, and responsibilities of the coordinating centre, steering committee, endpoint adjudication committee, data management team, and other individuals or groups overseeing the trial, if applicable (see Item 21a for data monitoring committee)	Methods and analysis - data monitoring and management
**Introduction**			
Background and rationale	6a	Description of research question and justification for undertaking the trial, including summary of relevant studies (published and unpublished) examining benefits and harms for each intervention	Introduction
	6b	Explanation for choice of comparators	Methods and analysis - the intervention
Objectives	7	Specific objectives or hypotheses	Introduction - aims and objectives
Trial design	8	Description of trial design including type of trial (eg, parallel group, crossover, factorial, single group), allocation ratio, and framework (eg, superiority, equivalence, noninferiority, exploratory)	Methods and analysis - design
**Methods: Participants, interventions, and outcomes**			
Study setting	9	Description of study settings (eg, community clinic, academic hospital) and list of countries where data will be collected. Reference to where list of study sites can be obtained	Methods and analysis - setting
Eligibility criteria	10	Inclusion and exclusion criteria for participants. If applicable, eligibility criteria for study centres and individuals who will perform the interventions (eg, surgeons, psychotherapists)	Methods and analysis - eligibility criteria

For peer review only - http://bmjopen.bmj.com/site/about/guidelines.xhtml

Interventions	11a	Interventions for each group with sufficient detail to allow replication, including how and when they will be administered	Methods and analysis - the intervention/ treatment as usual
	11b	Criteria for discontinuing or modifying allocated interventions for a given trial participant (eg, drug dose change in response to harms, participant request, or improving/worsening disease)	Ethics and dissemination - serious adverse events
	11c	Strategies to improve adherence to intervention protocols, and any procedures for monitoring adherence (eg, drug tablet return, laboratory tests)	Methods and analysis - the intervention
	11d	Relevant concomitant care and interventions that are permitted or prohibited during the trial	Methods and analysis - treatment as usual
Outcomes	12	Primary, secondary, and other outcomes, including the specific measurement variable (eg, systolic blood pressure), analysis metric (eg, change from baseline, final value, time to event), method of aggregation (eg, median, proportion), and time point for each outcome. Explanation of the clinical relevance of chosen efficacy and harm outcomes is strongly recommended	Methods and analysis - outcomes
Participant timeline	13	Time schedule of enrolment, interventions (including any run-ins and washouts), assessments, and visits for participants. A schematic diagram is highly recommended (see Figure)	Figure 1
Sample size	14	Estimated number of participants needed to achieve study objectives and how it was determined, including clinical and statistical assumptions supporting any sample size calculations	Methods and analysis - participants
Recruitment	15	Strategies for achieving adequate participant enrolment to reach target sample size	Methods and analysis - recruitment
**Methods: Assignment of interventions (for controlled trials)**			

Allocation:			
Sequence generation	16a	Method of generating the allocation sequence (eg, computer-generated random numbers), and list of any factors for stratification. To reduce predictability of a random sequence, details of any planned restriction (eg, blocking) should be provided in a separate document that is unavailable to those who enrol participants or assign interventions	Methods and analysis - randomisation
Allocation concealment mechanism	16b	Mechanism of implementing the allocation sequence (eg, central telephone; sequentially numbered, opaque, sealed envelopes), describing any steps to conceal the sequence until interventions are assigned	Methods and analysis - randomisation
Implementation	16c	Who will generate the allocation sequence, who will enrol participants, and who will assign participants to interventions	Methods and analysis - randomisation
Blinding (masking)	17a	Who will be blinded after assignment to interventions (eg, trial participants, care providers, outcome assessors, data analysts), and how	Methods and analysis - randomisation
	17b	If blinded, circumstances under which unblinding is permissible, and procedure for revealing a participant's allocated intervention during the trial	N/A
**Methods: Data collection, management, and analysis**			
Data collection methods	18a	Plans for assessment and collection of outcome, baseline, and other trial data, including any related processes to promote data quality (eg, duplicate measurements, training of assessors) and a description of study instruments (eg, questionnaires, laboratory tests) along with their reliability and validity, if known. Reference to where data collection forms can be found, if not in the protocol	Methods and analysis - data collection
	18b	Plans to promote participant retention and complete follow-up, including list of any outcome data to be collected for participants who discontinue or deviate from intervention protocols	Methods and analysis - data monitoring and management

Data management	19	Plans for data entry, coding, security, and storage, including any related processes to promote data quality (eg, double data entry; range checks for data values). Reference to where details of data management procedures can be found, if not in the protocol	Methods and analysis - data monitoring and management
Statistical methods	20a	Statistical methods for analysing primary and secondary outcomes. Reference to where other details of the statistical analysis plan can be found, if not in the protocol	Methods and analysis - data analysis
	20b	Methods for any additional analyses (eg, subgroup and adjusted analyses)	N/A
	20c	Definition of analysis population relating to protocol non-adherence (eg, as randomised analysis), and any statistical methods to handle missing data (eg, multiple imputation)	Methods and analysis - data analysis
**Methods: Monitoring**			
Data monitoring	21a	Composition of data monitoring committee (DMC); summary of its role and reporting structure; statement of whether it is independent from the sponsor and competing interests; and reference to where further details about its charter can be found, if not in the protocol. Alternatively, an explanation of why a DMC is not needed	Methods and analysis - data monitoring and management
	21b	Description of any interim analyses and stopping guidelines, including who will have access to these interim results and make the final decision to terminate the trial	Methods and analysis - data analysis
Harms	22	Plans for collecting, assessing, reporting, and managing solicited and spontaneously reported adverse events and other unintended effects of trial interventions or trial conduct	Ethics and dissemination - serious adverse events
Auditing	23	Frequency and procedures for auditing trial conduct, if any, and whether the process will be independent from investigators and the sponsor	N/A
**Ethics and dissemination**			

Research ethics approval	24	Plans for seeking research ethics committee/institutional review board (REC/IRB) approval	Ethics and dissemination - ethical approval
Protocol amendments	25	Plans for communicating important protocol modifications (eg, changes to eligibility criteria, outcomes, analyses) to relevant parties (eg, investigators, REC/IRBs, trial participants, trial registries, journals, regulators)	Ethics and dissemination - ethical approval
Consent or assent	26a	Who will obtain informed consent or assent from potential trial participants or authorised surrogates, and how (see Item 32)	Methods and analysis - recruitment
	26b	Additional consent provisions for collection and use of participant data and biological specimens in ancillary studies, if applicable	N/A
Confidentiality	27	How personal information about potential and enrolled participants will be collected, shared, and maintained in order to protect confidentiality before, during, and after the trial	Ethics and dissemination - confidentiality
Declaration of interests	28	Financial and other competing interests for principal investigators for the overall trial and each study site	Declarations - competing interests
Access to data	29	Statement of who will have access to the final trial dataset, and disclosure of contractual agreements that limit such access for investigators	Methods and analysis - data monitoring and management
Ancillary and post-trial care	30	Provisions, if any, for ancillary and post-trial care, and for compensation to those who suffer harm from trial participation	N/A

For peer review only - http://bmjopen.bmj.com/site/about/guidelines.xhtml

Dissemination policy	31a	Plans for investigators and sponsor to communicate trial results to participants, healthcare professionals, the public, and other relevant groups (eg, via publication, reporting in results databases, or other data sharing arrangements), including any publication restrictions	Ethics and dissemination – dissemination/ Methods and analysis – patient and public involvement
	31b	Authorship eligibility guidelines and any intended use of professional writers	Declarations – authors' contributions
	31c	Plans, if any, for granting public access to the full protocol, participant-level dataset, and statistical code	Data statement
**Appendices**			
Informed consent materials	32	Model consent form and other related documentation given to participants and authorised surrogates	Additional file 3
Biological specimens	33	Plans for collection, laboratory evaluation, and storage of biological specimens for genetic or molecular analysis in the current trial and for future use in ancillary studies, if applicable	N/A

*It is strongly recommended that this checklist be read in conjunction with the SPIRIT 2013 Explanation & Elaboration for important clarification on the items. Amendments to the protocol should be tracked and dated. The SPIRIT checklist is copyrighted by the SPIRIT Group under the Creative Commons "Attribution-NonCommercial-NoDerivs 3.0 Unported" license.

For peer review only

World Health Organization Trial Registration Data Set

Primary Registry and Trial Identifying Number	ISRCTN10004994
Date of Registration in Primary Registry	25/05/2018
Secondary Identifying Numbers	IRAS ID: 182553<br>REC reference: 15/LO/1453
Source(s) of Monetary or Material Support	The research is funded by the National Institute for Health Research (NIHR) Collaboration for Leadership in Applied Health Research and Care North Thames at Barts Health NHS Trust (NIHR CLAHRC North Thames).
Primary Sponsor	Camden and Islington NHS Foundation Trust
Secondary Sponsor(s)	N/A
Contact for Public Queries	Thomas Steare<br><br>+44 (0)20 7679 8192<br>thomas.steare.15@ucl.ac.uk<br>UCL Division of Psychiatry<br>6th Floor, Maple House<br>149 Tottenham Court Road<br>London<br>W1T 7NF
Contact for Scientific Queries	Prof Sonia Johnson<br><br>+44 (0)20 7679 9453<br>s.johnson@ucl.ac.uk<br>UCL Division of Psychiatry<br>6th Floor, Maple House<br>149 Tottenham Court Road<br>London<br>W1T 7NF
Public Title	ARIES: App to support Recovery in Early Intervention Services
Scientific Title	App to support Recovery In Early intervention Services (the ARIES study): feasibility trial of a supported self-management Smartphone application for psychosis
Countries of Recruitment	England
Health Condition(s) or Problem(s) Studied	First-episode psychosis
Intervention(s)	Self-management Smartphone app (My Journey 3)
Key Inclusion and Exclusion Criteria	Participant inclusion criteria

For peer review only

	1. Currently on the caseload of an Early Intervention Service and in contact with clinicians<br>2. Aged 16 or older<br>3. Have a diagnosis of psychosis<br>4. Own an Android Smartphone.<br><br>Participant exclusion criteria<br><br>1. Lack capacity to provide consent to take part in the study<br>2. Unable to communicate and understand English sufficiently to understand trial procedures and use the app<br>3. In the view of their EIS team, pose such a high risk to others that it would be unsafe to conduct research meetings even on NHS premises.
Study Type	Multi-centre randomised controlled feasibility trial
Date of First Enrolment	09/03/2017
Sample Size	40
Recruitment Status	Recruitment target met – no longer recruiting
Primary Outcome(s)	Relapse as indicated by admission to acute care (inpatient wards, crisis resolution teams, crisis houses and acute day services) during the 12-month follow-up period. Data on admissions to acute care during the trial period is collected from patient records at the 12-month follow-up.
Key Secondary Outcomes	1. Social outcomes are measured using The Social Outcomes Index (Priebe, Watzke, Hansson & Burns, 2008) at the study baseline meeting, at a 4-month follow-up meeting and at a 12-month follow-up meeting<br>2. Mental wellbeing is assessed using The Mental Health Confidence Scale (Carpinello et al., 2000) and The Warwick-Edinburgh Mental Well-Being Scale (NHS Health Scotland, University of Warwick & University of Edinburgh, 2007) at the study baseline meeting, at a 4-month follow-up meeting and at a 12-month follow-up meeting<br>3. Recovery in psychosis is assessed using The Process of Recovery Questionnaire (Neil et al., 2009) at the study baseline meeting, at a 4-month follow-up meeting and at a 12-month follow-up meeting<br>4. Quality of life and satisfaction with treatment is assessed using The DIALOG scale (Priebe et al., 2007) at the study baseline meeting, at a 4-month follow-up meeting and at a 12-month follow-up meeting<br>5. Positive, negative and general psychopathology symptoms are assessed using the PANSS (Kat et al., 1987) at the study

For peer review only

	baseline meeting, at a 4-month follow-up meeting and at a 12-month follow-up meeting<br>6. Participants' engagement with Early Intervention Services during the study period is obtained using the Service Engagement Scale (SES; Tait et al., 2002) completed by participants' clinicians at baseline and at the 12-month follow-up.<br>7. The following patient information is collected from patient records at baseline and one year after entry into the study:<br>7.1. Current diagnosis<br>7.2. Current care cluster<br>7.3. Care plan approach status<br>8. The usability and acceptability of My Journey 3 for service users and clinicians is assessed from semi-structured qualitative interviews conducted at the 4-month follow-up meeting.
Ethics Review	National Research Ethics Service Committee London - Brent, 02/10/2015, ref: 15/LO/1453. Amendment approved 29/07/2017.
Completion date	Study is ongoing

For peer review only

Service user participant consent form

Study Title: App to support Recovery In Early intervention Services (the ARIES study): Pilot randomised controlled trial of a self-management smartphone application

Principal Investigators: Professor Sonia Johnson and Professor David Osborn

Please Initial Each Box

1. I confirm that I have read and understood the Participant Information Sheet V5 dated 29/05/2017 for the above study and have had the opportunity to ask questions about the study. ☐
2. I understand that my participation is voluntary and that I am free to withhold personal information or to withdraw my participation at any time, without giving any reason, and without my medical care or legal rights being affected. ☐
3. I understand that if I choose to withdraw from the study that any data that I have already provided for the purposes of the research will be kept and used by the research team. ☐
4. I give permission for my General Practitioner (GP) and my Early Intervention team to be told I am participating in this study. ☐
5. I understand that relevant sections of my medical notes and data collected during the study may be looked at by regulatory authorities or from the NHS Trust, where it is relevant to my taking part in this research. I give permission for these individuals to have access to my records. ☐
6. I understand that I will be given a £20 gift as cash for my participation in each study assessment. ☐
7. I agree to the research team consulting NHS electronic records to investigate my diagnosis, medication, and mental health service use, and give them permission to do so even if I choose to no longer participate in the intervention, or they are not able to carry out further study interviews with me. ☐
8. I understand that in the event that I disclose information which may indicate new risk to myself or others, the researcher will be obliged to follow NHS Trust risk procedures that may require release of my personal data. ☐
9. I give permission for findings from the study to be written up for publication. Any publication will not identify me. ☐
10. I give permission to be audio recorded where required for the purposes of the study. I understand these audio-recordings will be transcribed and anonymised and audio recordings destroyed after the study. I give permission for direct quotations taken from this interview to be included in papers written for publication. Any quotation would not identify me. ☐
11. I give permission for the research team to collect data from the My Journey 3 app regarding the frequency, duration, and pattern of my use of it. I understand that no personal information will be collected from the app. ☐
12. I give permission for non-identifiable data to be shared with other research teams for research purposes. ☐

For peer review only

For peer review only - http://bmjopen.bmj.com/site/about/guidelines.xhtml

13. I agree to take part in this study. ☐

________________________	______________	________________________
Name of participant	Date	Signature

________________________	______________	________________________
Name of Researcher taking consent	Date	Signature

For peer review only

For peer review only - http://bmjopen.bmj.com/site/about/guidelines.xhtml

BMJ Open

The App to support Recovery in Early Intervention Services (ARIES) Study: Protocol of a feasibility randomised controlled trial of a self-management Smartphone application for psychosis

Journal:	*BMJ Open*
Manuscript ID	bmjopen-2018-025823.R1
Article Type:	Protocol
Date Submitted by the Author:	30-Oct-2018
Complete List of Authors:	Steare, Thomas; University College London, Division of Psychiatry; Camden and Islington NHS Foundation Trust,<br>O'Hanlon, Puffin; University College London, Division of Psychiatry<br>Eskinazi, Michelle; University College London, Division of Psychiatry<br>Osborn, David; UCL, Faculty of Brain Sciences; Camden and Islington NHS Foundation Trust<br>Lloyd-Evans, Brynmor ; UCL, Division of Psychiatry; Camden and Islington NHS Foundation Trust<br>Jones, Rebecca; UCL, Division of Psychiatry<br>Rostill, Helen; Surrey and Borders Partnership NHS Foundation Trust<br>Amani, Sarah; NHS England, Early Intervention in Psychosis Programme (South of England)<br>Johnson, Sonia; UCL, Division of Psychiatry ; Camden and Islington NHS Foundation Trust
**Primary Subject Heading**:	Mental health
Secondary Subject Heading:	Mental health
Keywords:	Schizophrenia & psychotic disorders < PSYCHIATRY, MENTAL HEALTH, PSYCHIATRY

SCHOLARONE™
Manuscripts

TITLE PAGE

Title: The App to support Recovery in Early Intervention Services (ARIES) Study: Protocol of a feasibility randomised controlled trial of a self-management Smartphone application for psychosis

Authors:

Thomas Steare

Division of Psychiatry, University College London, London, UK

R&D Department, Camden and Islington NHS Foundation Trust, London, UK

Puffin O'Hanlon

Division of Psychiatry, University College London, London, UK

Michelle Eskinazi

Division of Psychiatry, University College London, London, UK

David Osborn

Division of Psychiatry, University College London, London, UK

R&D Department, Camden and Islington NHS Foundation Trust, London, UK

Brynmor Lloyd-Evans

Division of Psychiatry, University College London, London, UK

R&D Department, Camden and Islington NHS Foundation Trust, London, UK

Rebecca Jones

Division of Psychiatry, University College London, London, UK

Helen Rostill

Surrey and Borders Partnership NHS Foundation Trust, Leatherhead, Surrey, UK

For peer review only

Sarah Amani

Senior Programme Manager, EIP Programme (South of England), NHS England, Oxford, Oxfordshire, UK

Sonia Johnson (Corresponding author)

Address: UCL Division of Psychiatry, 6th Floor, Wing B, Maple House, 149 Tottenham Court Road, London W1T 7NF

Division of Psychiatry, University College London, London, UK

R&D Department, Camden and Islington NHS Foundation Trust, London, UK

s.johnson@ucl.ac.uk

02076799453

Trial Sponsor: Camden & Islington NHS Foundation Trust. sponsor.noclor@nhs.net

Study start date (first participant recruited): 09 March 2017

Study end date: 31 March 2019

Keywords: Psychosis, Self-management, Smartphone, Feasibility study, Randomised controlled trial

Word count: 4,546

For peer review only

ABSTRACT

Introduction: Mental health interventions delivered through digital technology have potential applications in promoting recovery and improving outcomes among people in the early stages of psychosis. Self-management approaches are recommended for the treatment of psychosis and could be delivered via applications (apps) installed on Smartphones to provide low-cost accessible support. We describe the protocol for a feasibility trial investigating a self-management Smartphone app intervention for adults using Early Intervention in Psychosis (EIP) services.

Methods and analysis: In this feasibility randomised controlled trial, 40 participants will be recruited from EIP services in London and Surrey. Twenty participants will be randomised to receive a supported self-management Smartphone app (My Journey 3) plus Treatment As Usual (TAU), while the other 20 participants will receive TAU only. The primary objective of this study is to evaluate the feasibility of conducting a full-scale trial of this intervention in EIP services. Participant data will be collected at baseline and at two follow-up assessments conducted four months and 12 months post-baseline. Analysed outcome measures will include relapse of psychosis (operationalised as admission to a hospital or community acute alternative), mental health and wellbeing, recovery, quality of life and psychopathology. Semi-structured interviews with participants and EIP service clinicians will additionally explore experiences of using My Journey 3 and participating in the trial and suggestions for improving the intervention.

Ethics and dissemination: The ARIES study has been reviewed and approved by the National Research Ethics Service Committee London – Brent (Research Ethics Committee reference: 15/LO/1453). The findings of this study will be disseminated through peer-reviewed scientific journals and conferences, magazines and web publications.

For peer review only

Trial registration number: ISRCTN10004994

ARTICLE SUMMARY

Strengths and limitations of this study

- The app has been well developed prior to the feasibility trial, including substantial stakeholder input to the development of the intervention at all stages, an initial period of testing in clinical settings, and refinement of the current version through lab and field testing.
- A range of types of data contribute to our assessment of feasibility, including psychometrically robust outcome measures, qualitative data on experiences of use, and usage data.
- The trial will only recruit Android Smartphone users as My Journey 3 is not as yet compatible with iOS or other Smartphone operating systems; this will limit sample representativeness.
- Assessor and participant blinding to group allocation will not be feasible in this trial. This is a feasibility study, thus not powered to draw conclusions regarding the effectiveness of the intervention.

For peer review only

INTRODUCTION

Psychosis is associated with significant costs at individual-, family- and society-level.[1-3] The implementation of Early Intervention in Psychosis (EIP) services across the United Kingdom has been associated with improved outcomes,[4,5] however substantial challenges remain. Long-term follow-up studies have shown that relapse rates in the early course of psychosis remain high.[6] Furthermore functional recovery is often not attained following treatment.[7]

Thus there is a need to increase and sustain benefits from EIP services. An approach with demonstrated efficacy in improving functional recovery and reducing relapse in psychosis is supported self-management.[8] Self-management has been designed to empower people as active agents in their own recovery by enabling them to develop skills such as recognising and monitoring symptoms and early warning signs of relapse, identifying and avoiding stressors, and using effective coping strategies.[9] Self-management is associated with significant benefits including reduced distress, improved medication adherence and reduced number of hospitalizations.[10-12] Relapse prevention work has long been proposed as a key element in EIP,[13] and establishing and working towards recovery goals is widely advocated in EIP services.[14] However, well-evaluated tools and methods are currently not available to support widespread implementation of self-management approaches in EIP services.

Over recent years there has been growing interest in the development of innovative technologies in health-care due to their potential to improve accessibility, efficacy, quality and cost-effectiveness of treatment.[15] Due to rapid advancements in mobile phone technology, it is possible to deliver clinical interventions via applications (apps) installed on Smartphones (mobile phones with computational capacities). Smartphones are widely available in the UK with over 75% of adults owning one.[16] They are often carried on the person with high

For peer review only

accessibility to the internet and are therefore a suitable device to provide time-unlimited interventions in almost any location.[17]

Recent evidence suggests that people with psychosis are adopting digital technology in a similar way to the general population,[18-20] and that they are interested in using mental health interventions delivered via Smartphones.[21,22] A systematic review has found that interventions delivered through apps or text messaging on Smartphones are acceptable and feasible for people with psychosis and may support recovery.[23] Smartphone apps based on self-management principles have shown promise in a wide range of long-term health conditions,[24] and are a potential way to deliver accessible low-cost support to adults with psychosis.

Although emerging research suggests that Smartphone apps hold promise in delivering effective interventions to adults with psychosis, the evidence base is under-developed in comparison to more common mental disorders.[25] To date only one randomised controlled trial (RCT) of a psychosocial Smartphone app used in EIP services has been published.[26] In this proof-of-concept trial, 36 adults accessing EIP services were randomised to use a cognitive-behavioural therapy informed Smartphone app (Actissist) that aims to encourage active self-management, or a symptom-monitoring app which was classed as the control condition. Actissist was found to be acceptable, feasible and safe for adults accessing EIP services. Although not powered to find an effect, the study suggests that Actissist may confer benefits to users' outcomes over and above a passive symptom-monitoring app. Participants however only had access to the app for 12 weeks, were followed up over a relatively short timeframe (22 weeks) and significant mental health outcomes such as relapse were not measured. This work has been conducted in parallel with our study, and we anticipate both programmes of work will contribute to developing an evidence base as to whether Smartphone

For peer review only

apps that promote self-management can improve outcomes in first-episode psychosis and reduce healthcare costs.

Aims and objectives

The aim of the ARIES study is to examine the feasibility of conducting a full-scale trial of a clinician supported self-management Smartphone app for adults accessing EIP services. The feasibility trial will aim:

1. To identify whether a self-management Smartphone app is acceptable to use and feasible to support in EIP services in the context of a research study, and to identify any necessary modifications to the intervention content and design, or to its delivery in EIP services.
2. To test the feasibility and acceptability of trial procedures for a definitive trial.
3. To test procedures for evaluating intervention engagement and participant outcomes.

METHODS AND ANALYSIS

Design

The study is a feasibility RCT comparing a clinician supported self-management Smartphone app (My Journey 3), in addition to Treatment As Usual (TAU), to a control group receiving TAU only. The design as described here adheres to the Standard Protocol Items: Recommendations for Interventional Trials (SPIRIT).[27] A copy of the SPIRIT checklist is provided as Additional file 1. Relevant items from the World Health Organization Trial Registration Data Set are detailed in Additional file 2.

Setting

The trial will take place in six EIP services across three NHS Foundation Trusts in England; Camden and Islington, East London, and Surrey and Borders Partnership. All

For peer review only

participating EIP services provide care coordination to service users, access to a psychiatrist and psychiatric medication and psychosocial interventions, aiming to conform to the current UK model for EIP services.[28] None of the participating EIP services offer Smartphone apps or any other digital interventions as part of routine care, nor are structured self-management tools consistently available in these teams. A list of participating sites is available from the authors.

Participants

Forty service users will be recruited from the participating EIP services. Assuming a conservative estimate of a 40% attrition rate, the study should feature 12 completer participants in each group as recommended for feasibility and pilot trials.[29] The proposed sample size will therefore be sufficient to establish the acceptability of My Journey 3 and the feasibility of trial procedures.

Eligibility criteria

Inclusion criteria

- Aged 16 or older
- Have experienced at least one episode of psychosis
- Currently on the caseload of an EIP service and in contact with clinicians
- User of a Smartphone with an Android operating system.

Exclusion criteria

- Lack of capacity to provide informed consent to participate in the trial
- Inability to communicate and understand English sufficiently to understand trial procedures and use My Journey 3

For peer review only

- In the view of their EIP service, poses such a high risk to others that it would be unsafe to conduct research meetings even on NHS premises.

Criteria are deliberately broad in order to reach conclusions generalizable to a wide range of EIP service users. Due to limited resources My Journey 3 has been developed for one Smartphone operating system (Android) at this stage of testing. Nearly half of Smartphones used in the UK utilise Android operating systems.[30] There is also emerging evidence that Android may be the Smartphone operating system most often used by adults with severe mental illness.[21] Resources are not currently available to deliver My Journey 3 in languages other than English.

Recruitment

An overview of the recruitment procedure is displayed in the flow diagram (figure 1). Clinicians will identify EIP service users who appear potentially eligible for the trial. They will then make initial contact, explain the trial and ask if the potential participant is willing to be contacted by a researcher. If a potential participant is eligible and interested in taking part, a researcher will contact them to further explain the trial and to arrange a face-to-face meeting. At this meeting the researcher will provide the trial information sheet, confirm eligibility, invite any questions about participating and assess the service user's capacity to provide informed consent. Written consent to participate will be obtained using a consent form (Additional file 3) prior to undertaking the baseline assessment at the same meeting. Participants will be notified that they will be free to withdraw from the study at any time-point. Prior to study recruitment, researchers will undertake training in how to assess mental capacity and take informed consent.

For peer review only

To minimize loss to follow-up, participants will be asked to give their preferred contact details, and for their permission for the researcher to contact a nominated close other or EIP service clinician whom could contact the participant if the researcher is unable to contact the participant directly.

Randomisation

At a separate time-point following the baseline assessment meeting participants will be randomly allocated in a 1:1 ratio to an intervention group where they will have access to My Journey 3 during the trial (n=20) or to a control group (n=20). Randomisation will be conducted by an independent statistician. The allocation sequence will be concealed from the researcher, who will be blind when recruiting participants and conducting baseline assessments. An independent researcher will hold on to the allocation list and disclose participants' allocation to the trial researcher after the completion of the baseline assessments. Participants will then be informed of their allocation by the trial researcher. Participants cannot be blinded to their group allocation given the nature of the trial intervention and control group. As a single researcher will carry out most of the data collection, it is not practical for the group allocation of participants to be concealed from the research team in this feasibility study.

The intervention

Development

My Journey 3 has been developed through a series of iterations. The first version of My Journey was designed by Surrey and Borders Partnership NHS Foundation Trust (led by Sarah Amani), after local EIP service users consulted about their care suggested there would be significant benefits from a Smartphone app that could be used for appointment and medication reminders, to track their mood and to share their recovery progress with EIP staff or carers. In

For peer review only

2011 a project group consisting of EIP service users, NHS clinicians, a pharmacist and a NHS manager was formed to drive the development of the My Journey app. The following year a prototype of My Journey was tested by 20 EIP service users to further inform development of the app. Since April 2013 this version of My Journey has been available for download from Google Play (the official app store for Android Smartphones).

The original version of My Journey contains generic advice on what to do if certain clinical difficulties arise, but does not allow personalised relapse prevention planning or recovery goal tracking. User feedback suggested that this was a limitation. In the current study we have collaborated with digital health experts, EIP service clinicians and adults with lived experience of psychosis to adapt existing paper-and-pen self-management intervention components – in routine use in NHS services - to be suitable for delivery in an app format [31,32]. In creating the product specification of My Journey 3 we have incorporated these self-management components with functions from the original My Journey. Technical development of My Journey 3 has been led by MyOxygen (https://myoxygen.uk/), a private app development company based in the UK.

Following the initial development of My Journey 3, it has been refined in response to two phases of preliminary testing. In the first phase six volunteer EIP services users participated in usability lab tests with My Journey 3 on their own Android Smartphone. Usability testing featured "think aloud" tests where participants completed set tasks using My Journey 3 while providing a continuous commentary on their thoughts. This method was used to highlight design and usability issues and users' immediate reactions to the app. Individual interviews were also conducted to explore participants' perceptions of the ease of use of My Journey 3, concerns they might have and suggestions for improvements. My Journey 3 was modified to reflect the findings at this stage.

For peer review only

In the second phase a further six volunteer service users trialled My Journey 3 on their own Android Smartphone during a one-month field study, with EIP service clinicians asked to support their clients' use of the app during routine appointments. Following the field study, individual interviews were conducted with service user participants to explore how they used My Journey 3 and how it could be improved. Further interviews with EIP service clinicians explored their experience of supporting their client with My Journey 3. Based on these findings My Journey 3 was updated again prior to the feasibility trial.

Intervention outline

My Journey 3 is a Smartphone app that has been designed to be used alongside EIP service care and with the support of clinicians. My Journey 3 aims to develop and support users' illness self-management skills to help facilitate recovery from first-episode psychosis. The My Journey 3 home screen can be seen in figure 2.

Three main components of My Journey 3 have been directly taken from the original app and updated. Information regarding psychosis, mental health and mental health services is provided in My Journey 3 through links to relevant NHS and voluntary sector websites and through videos of personal recovery stories. To facilitate symptom recognition and monitoring My Journey 3 also features a self-monitoring tool and symptom tracker where users can monitor 14 different symptoms and 3 lifestyle behaviours. Advice to help manage symptoms is provided after completing the symptom tracker, and a graphical summary of symptom severity over time is displayed. Users also have access to a pill tracker where they can log whether they have taken their psychiatric medication. The pill tracker features a daily alert at a pre-set time to remind users to input if they have taken their medication.

For peer review only

In designing My Journey 3 we have also drawn on evidenced-based self-management interventions to add structured intervention components focused on recovery planning and relapse prevention.[31,32] These additions allow for users to interactively:

- Identify strategies and coping resources that they find useful in maintaining wellbeing
- Set and track progress towards personal recovery goals
- Identify personal early warning signs of relapse and strategies and coping mechanisms to put in place should they experience these
- Create a "relapse plan", an action plan to follow in times of crisis in order to avoid or attenuate relapse

My Journey 3 has been designed to be used by EIP service users in collaboration with clinicians. Clinicians can input relevant information to sections such as the relapse prevention plans and, with training, can provide assistance with the app. It is also suitable for independent use: the developers' aspiration is for the app to be initially used in collaboration with clinicians, but for it then to support users' self-management following discharge from EIP services.

My Journey 3 features weekly discrete notifications that appear on the users' Smartphone interface to encourage engagement with the app. Users also have the opportunity to set activity reminders that notify the user to engage in coping strategies and take part in pre-identified activities to promote wellbeing.

My Journey 3 also features a sharing functionality for users who wish to share their data with clinicians, family members, friends or other trusted third parties. This uses built-in sharing functionality of the user's Smartphone, such as e-mail. Participants will have control of who they choose to share their data with at all times. The research team will have no responsibility for providing clinical care if any My Journey 3 data shared by a participant to a

For peer review only

third party indicates a decline in their mental health. Third parties such as EIP service clinicians and carers would be expected to act as appropriate.

Delivery of the intervention

Each participant in the intervention group will take part in an individual training session with their supporting EIP service clinician and a researcher within six weeks of the initial consent meeting. During each training session participants will download My Journey 3 on to their Smartphone. The researcher will then give a demonstration of the app and its main functions. Participants will have the opportunity to practice using My Journey 3 and to ask questions. To facilitate use during the trial participants will be asked to input appropriate information in to the main functions of My Journey 3. The researcher will encourage participants and supporting EIP service clinicians to discuss recovery goals and relapse prevention plans in following routine appointments and then to input them into My Journey 3. If supporting clinicians leave the participants' EIP service the researcher will arrange a meeting to introduce any new clinicians that have clients using My Journey 3, to the intervention and how they can support service users that access to the app. Participants will have access to My Journey 3 from the training session till the 12-month time-point. Researcher support with My Journey 3 will be limited to the initial installation and technical support as needed during the trial period. Participants in the intervention group will be free to withdraw from using My Journey 3, or decline the installation of it on to their Smartphone, without any impact on their study participation.

Control group

Participants in the control group (n=20) will receive TAU that will be unaffected by their participation.

For peer review only

Treatment As Usual

TAU for service users attending EIP services typically involves regular meeting with a care co-ordinator, support from multi-disciplinary clinicians and access to a psychiatrist, psychiatric medication, and a range of psychological interventions.

Data collection

The participant timeline is summarised in table 1. All participants will be asked to complete self-report questionnaires during a structured assessment with a researcher at three time points; baseline (prior to randomisation), 4-months post baseline and 12-months post baseline. Participants in the intervention group will also be invited to complete an audio-recorded interview with a researcher during the 4-month follow-up assessments. Before arranging each assessment the researcher will check with any clinicians in contact with the service users that the participant does not pose a risk to others and that it would be safe to conduct a research meeting. If there is an identified risk too serious for a meeting on NHS premises the participant will not be met for the assessment at that time-point, with the reason for not arranging a meeting documented. If any major concerns regarding participants' risk or well-being arise during interactions with the researcher, this would be communicated to the appropriate EIP service.

Prior to the completion of each assessment participants will be provided with a trial information sheet. Their capacity to give informed consent will be assessed and their consent documented in writing. Participants will receive 20 pounds as a token of thanks for completing each assessment. Participants that have been discharged from EIP services during the trial will still be invited to attend assessments.

Once the recruitment target for the study has been met, the researcher will contact the appropriate administrators or informatics team within each NHS trust to arrange the collection

For peer review only

of participant data from patient records. The researcher will also arrange for data to be collected from patient records one year later.

To test procedures for evaluating engagement with the intervention, data regarding My Journey 3 use will be collected throughout the trial period for all participants in the intervention group. My Journey 3 will automatically upload encrypted usage data to a secure trial server when the user has internet access on their Smartphone.

For peer review only

Table 1. Timeline of participant enrolment, interventions, assessments and patient records data collection

**Timepoint**	**Enrolment**	**Baseline**	**Allocation**	**MJ3 training session***	**4 month follow-up**	**12 month follow-up**
**Enrolment:**						
Eligibility screen	X					
Informed consent		X				
Randomisation			X			
**Intervention:**						
My Journey 3 (intervention group)				←	—	→
TAU (all participants)			←	—	—	→
**Assessments:**						
PANSS		X			X	X
Sociodemographic information		X			X	X
Clinical service use		X			X	X
Social Outcomes Index		X			X	X
Mental Health Confidence Scale		X			X	X
The Process of Recovery Questionnaire		X			X	X
WEMWBS		X			X	X
The DIALOG Scale		X			X	X
Service Engagement Scale (completed by EIP service clinicians)		X				X
Qualitative interviews (with participants in the intervention group and supporting clinicians)					X	
**Patient records data (from previous 12 months to time point):**						
Number of admissions to acute mental health services		X				X
Number of compulsory admissions to acute mental health services		X				X
Total number of days in acute care		X				X
Number of kept appointments with community mental health services		X				X
Number of missed appointments with community mental health services		X				X
Primary ICD-10 diagnosis		X				X
Most recent care cluster		X				X
Care Programme Approach status		X				X

MJ3 My Journey 3, *WEMWBS* Warwick-Edinburgh Mental Well-being Scale, *PANSS* Positive and Negative Syndrome Scale
*Participants in the intervention group only.

Measures

Data from patient records

The following data will be collected from patient records at baseline and the 12 month time-point:

a. Most recent clinical diagnosis as recorded in patient records using the ICD-10 (Internal Classification of Diseases: 10th Revision) classification.
b. Most recent care cluster (a classification of mental health service users based on their needs, used in the NHS).
c. Whether participants are subject to a care programme approach (NHS mental health services' case management model).
d. Service use during the previous 12 months measured at baseline and 12-months post study entry. Data will include history of use of acute mental health services, inpatient admissions and compulsory admissions and the number of kept and missed appointments with mental health services. Relapse of psychosis, the proposed primary outcome for a fully powered RCT, will be operationalised as participant admission to an acute mental health service (inpatient psychiatric wards, crisis houses, crisis resolution teams and acute day care services).

My Journey 3 usage data

The data collected will be a record of each time the user opens My Journey 3, whether this was in response to a prompt, and which components they use. Usage data will not include participants' text input or responses to self-rated questions, and will therefore not give any information regarding the mental health of users. Usage data will be solely used to assess the acceptability of My Journey 3 and user engagement with it.

For peer review only

Self-report questionnaires

The following are measured as potential secondary outcomes for a future fully-powered RCT;

Service use measures over 1 year of follow-up

1. Engagement with EIP services as measured by the Service Engagement Scale (SES),[33] a 14-item questionnaire that measures engagement for four different dimensions; availability, collaboration, help-seeking and treatment adherence. The SES will be completed by EIP service clinicians, such as participants' care co-ordinators, at baseline and at the 12-month time-point.

Measures at baseline, 4-month and 12-month follow-up assessments

1. Psychotic symptoms and general psychopathology, measured by the Positive and Negative Syndrome Scale (PANSS), a 30-item scale that yields three separate scores on positive symptoms, negative symptoms and general psychopathology.[34] To inform the PANSS a trained researcher will conduct clinical interviews with participants at each assessment.
2. Social outcomes, rated by the Social Outcomes Index (SIX),[35] a 6-item index of social outcomes and circumstance.
3. Mental health-related self-efficacy, measured by the Mental Health Confidence Scale (MHCS),[36] a 16-item self-report scale of service users' confidence in their ability to cope with stressful or difficult events.
4. Self-rated recovery measured by the Questionnaire about the Process of Recovery (QPR),[37] a 22-item measure, yielding a total score and subscale scores for intrapersonal and interpersonal recovery factors.

For peer review only

5. Mental wellbeing, rated by total score on The Warwick-Edinburgh Mental Well-Being Scale (WEMWBS),[38] a 14-item self-report scale of mental wellbeing.
6. Subjective quality of life and satisfaction with treatment measured by The DIALOG scale,[39] an 11-item self-report scale.
7. Sociodemographic characteristics including age, gender and ethnicity. We will also collect data regarding accommodation and living situation, employment status, educational attainment and Smartphone use, including use of other mental health apps.

Qualitative interviews

Interviews with participants in the intervention group will follow a topic guide and will explore their experience of using My Journey 3, including:

- The usability and acceptability of My Journey 3
- Positives and negative aspects of My Journey 3
- Impact of My Journey 3 on their life
- Facilitators and barriers to using My Journey 3
- Views on the training session
- Views on the support they received from their clinician in using My Journey 3

EIP service clinicians who have been supporting participants with My Journey 3 will also be asked to complete an audio-recorded interview close to the time the participant completes the four-month follow-up assessment. Written consent to take part will be confirmed beforehand, with an information sheet provided. Clinicians will be asked to provide demographic data prior to undertaking the interview. The interview will follow a topic guide and will focus on:

- Positives and negative aspects of My Journey 3
- The experience of supporting clients with My Journey 3

For peer review only

- Facilitators and barriers to providing support and to the incorporation of My Journey 3 in clinical management
- Views on the training session

Data analysis

No pre-specified criteria have been set for establishing the acceptability of My Journey 3 or the feasibility of trial procedures. The acceptability of My Journey 3 for EIP service users and clinicians will be determined from feedback given from the qualitative interviews and the level of participants' My Journey 3 use indicated from the app usage data. Trial feasibility will be assessed from reviewing recruitment rates, drop-out rates and intervention enrolment and use during the trial period. These will be reviewed by the study team and discussed with stakeholders at a final dissemination event to decide on next steps.

Quantitative analysis

We will report rates of recruitment and retention in the trial, and, for the intervention group, the level of usage of My Journey 3 during the trial. The demographic and clinical characteristics of participants at baseline will be summarised separately for each study group using descriptive statistics.

To pilot the methods of analysis for a full-scale trial we will use an intention-to-treat approach using data from all randomised participants. The effect of My Journey 3 on relapse (the proposed primary outcome for a fully-powered RCT) during the 12 month trial period will be estimated using logistic regression. The effect of the intervention on continuous outcome measures (SIX, QPR, WEMWBS, DIALOG scale, PANSS and SES) will be estimated using linear regression adjusting for the baseline measure of the outcome in question. Results will be summarised using effect estimates and 95% confidence intervals only. No interim analyses are planned.

For peer review only

Qualitative analysis

To assess the acceptability of My Journey 3 interview data with participants in the intervention group and supporting EIP service clinicians will be analysed based on The Theoretical Framework of Acceptability for health care system interventions.[40] Analyses will be conducted collaboratively by a group of researchers.

Patient and Public Involvement

Independent advice will be sought from a trial steering group. Members will include researchers with expertise in developing and testing digital health interventions, EIP service clinicians, people with lived experience of first-episode psychosis and carers. Steering group members with lived experience of psychosis were consulted on the first paper prototypes of My Journey 3 and on the research protocol. The version of My Journey 3 tested in the current feasibility trial has been developed based on EIP service users' feedback from the usability tests and field study. Participants will be offered a summary of the research findings at the end of the study.

Data monitoring and management

A secure password-protected trial database will be developed and managed to store all quantitative data using SPSS software V.23, and will feature non-identifiable trial IDs only. Data entry of participants' questionnaires will be primarily undertaken by the trial researcher, with a random sample checked by other research team members. Anonymised electronic interview transcripts will be checked for accuracy and stored using NVivo for Windows (QSR International Pty Ltd V.11, 2016). After the trial, all data will be archived securely at University College London.

Due to the small sample size of the study a data monitoring committee is not planned, but the steering group will advise if one is later needed.

For peer review only

ETHICS AND DISSEMINATION

Ethical approval

Ethical approval has been obtained from the London Brent National Research Ethics Service Committee (Research Ethics Committee reference: 15/LO/1453), which has approved all amendments to protocol. Future protocol modifications will be submitted for approval to the research ethics committee and communicated to the study sponsor, site principle investigators, participating NHS trusts and participants. The current protocol in use is V.9, 29 July 2017.

Confidentiality

Participant data will be accessed by the research team only. Consent forms and data collection forms will be securely stored in locked cabinets at University College London. Data collection forms will not feature participants' names but a unique trial ID that could not be linked to participants by anyone outside the research team. Consent forms that identify participants will be kept separately from data collection forms.

Password-protected electronic data will be stored on the secure IT network at University College London. App usage data collected whilst using My Journey 3 will be anonymised and encrypted and will not contain personal user information.

Serious adverse events

Serious adverse events such as hospital admissions and death reported to the trial team will be reviewed by the Chief Investigator. Identified adverse events assessed as trial-related will be reported to the trial sponsor.

For peer review only

Dissemination

Results will be disseminated through scientific publications, and to a wider audience via magazines and web publications.

For peer review only

Acknowledgements: The ARIES research team are grateful to their software collaborators MyOxygen for their technical development and hosting of My Journey 3 and to Ali Mousa for his valuable contribution to the development of the original My Journey app. We are grateful to Max Birchwood for his permission to incorporate ‘Back in the Saddle’ in to My Journey 3. We are grateful to Rachel Perkins for her permission to adapt the Personal Recovery Plan resource and incorporate in to My Journey 3.

Author Contributions: The trial design was developed by SJ, DO, BLE and PO. SA, HR, PO and ME have led on the development of the intervention. RJ has advised on the statistical analysis. SJ is the Chief Investigator, based at University College London, DO the co-Chief Investigator, and TS the project manager. All authors have contributed and approved this manuscript.

Funding: The research is funded by the National Institute for Health Research (NIHR) Collaboration for Leadership in Applied Health Research and Care North Thames at Barts Health NHS Trust (NIHR CLAHRC North Thames). The views expressed in this article are those of the authors and not necessarily those of the NHS, the NIHR or the Department of Health and Social Care. SJ, DO and BLE are supported by the NIHR Mental Health Research Policy Unit, the NIHR Collaboration for Leadership in Applied Health Research and Care (CLAHRC) North Thames and the UCLH Biomedical Research Centre.

Data statement: The datasets generated during and/or analysed during the current study will be made available two years after the trial end.

Competing interests: None declared.

Legends:

Figure 1. SPIRIT flow diagram outlining the phases of the ARIES feasibility trial.

For peer review only

Figure 2. The My Journey 3 home screen, which is seen by the user when accessing the app on their Smartphone.

For peer review only

REFERENCES

[1] Addington J, Young J, Addington D. Social outcome in early psychosis. Psychol Med. 2003;33:1119-24.

[2] Addington J, Coldham EL, Jones B, Ko T, Addington D. The first episode of psychosis: the experience of relatives. Acta Psychiat Scand. 2003;108:285-9.

[3] The Schizophrenia Commission. The abandoned illness: a report from the Schizophrenia Commission. London: Rethink Mental Illness. 2012.

[4] Bird V, Premkumar P, Kendall T, Whittington C, Mitchell J, Kuipers E. Early intervention services, cognitive–behavioural therapy and family intervention in early psychosis: systematic review. Brit J Psychiat. 2010;197:350-6.

[5] Tsiachristas A, Thomas T, Leal J, et al. Economic impact of early intervention in psychosis services: results from a longitudinal retrospective controlled study in England. BMJ Open 2016;6:e012611. doi: 10.1136/bmjopen-2016-012611

[6] Alvarez-Jiménez M, Parker AG, Hetrick SE, McGorry PD, Gleeson JF. Preventing the second episode: a systematic review and meta-analysis of psychosocial and pharmacological trials in first-episode psychosis. Schizophrenia Bull. 2009;37:619-30.

[7] Menezes NM, Arenovich T, Zipursky RB. A systematic review of longitudinal outcome studies of first-episode psychosis. Psychol Med. 2006;36:1349-62.

[8] Mueser KT, Deavers F, Penn DL, Cassisi JE. Psychosocial treatments for schizophrenia. Annu Rev Clin Psycho. 2013;9:465-97.

[9] Mueser KT, Meyer PS, Penn DL, Clancy R, Clancy DM, Salyers MP. The Illness Management and Recovery program: rationale, development, and preliminary findings. Schizophrenia Bull. 2006;32:S32-43.

For peer review only

[10] Mueser KT, Corrigan PW, Hilton DW et al. Illness management and recovery: A review of the research. Psychiat Serv. 2002;53:1272-84.

[11] Cook JA, Copeland ME, Jonikas JA, et al. Results of a randomized controlled trial of mental illness self-management using Wellness Recovery Action Planning. Schizophrenia Bull. 2011;38:881-91.

[12] Färdig R, Lewander T, Melin L, Folke F, Fredriksson A. A randomized controlled trial of the illness management and recovery program for persons with schizophrenia. Psychiat Serv. 2011;62:606-12.

[13] Birchwood M, Spencer E, McGovern D. Schizophrenia: early warning signs. Advances in Psychiatric Treatment. 2000;6:93-101.

[14] Slade M. Personal Recovery and Mental Illness: A Guide for Mental Health Professionals. Cambridge: Cambridge University Press; 2009. (Values-Based Practice).

[15] Department of Health. Closing the Gap: Priorities for Essential Change in Mental Health. 2014, https://www.gov.uk/government/uploads/system/uploads/attachment_data/file/281250/Closing_the_gap_V2_-_17_Feb_2014.pdf (accessed August 2018)

[16] Ofcom. The Communications Market Report. London, UK: Ofcom; 2018

[17] Proudfoot J. The future is in our hands: the role of mobile phones in the prevention and management of mental disorders. Aust NZ J Psychiat. 2013;47:111-3.

[18] Firth J, Cotter J, Torous J, Bucci S, Firth JA, Yung AR. Mobile phone ownership and endorsement of "mHealth" among people with psychosis: a meta-analysis of cross-sectional studies. Schizophrenia Bull. 2015;42:448-55.

For peer review only

[19] Robotham D, Satkunanathan S, Doughty L, Wykes T. Do We Still Have a Digital Divide in Mental Health? A Five-Year Survey Follow-up. J Med Internet Res. 2016;18:e309.

[20] Naslund JA, Aschbrenner KA, Bartels SJ. How people with serious mental illness use smartphones, mobile apps, and social media. Psychiatr Rehabil J. 2016;39:364-367.

[21] Ben-Zeev D, Kaiser SM, Brenner CJ, Begale M, Duffecy J, Mohr DC. Development and usability testing of FOCUS: A smartphone system for self-management of schizophrenia. Psychiatr Rehabil J. 2013;36:289-296.

[22] Aref-Adib G, O'Hanlon P, Fullarton K, et al. A qualitative study of online mental health information seeking behaviour by those with psychosis. BMC Psychiatry. 2016;16:232.

[23] Bonet L, Izquierdo C, Escartí MJ, et al. Use of mobile technologies in patients with psychosis: A systematic review. Rev Psiquiatr Salud Ment. 2017;10:168-78.

[24] Whitehead L, Seaton P. The effectiveness of self-management mobile phone and tablet apps in long-term condition management: a systematic review. J Med Internet Res. 2016;18:e97.

[25] O'Hanlon P, Aref-Adib G, Fonseca A, et al. Tomorrow's world: current developments in the therapeutic use of technology for psychosis. BJPsych Advances. 2016;22:301-10.

[26] Bucci S, Barrowclough C, Ainsworth J, et al. Actissist: Proof-of-Concept Trial of a Theory-Driven Digital Intervention for Psychosis. Schizophrenia Bull. 2018; sby032.

[27] Chan A-W, Tetzlaff JM, Gøtzsche PC, et al. SPIRIT 2013 explanation and elaboration: guidance for protocols of clinical trials. BMJ. 2013;346:e7586

[28] National Institute for Health and Care Excellence. Implementing the Early Intervention in Psychosis Access and Waiting Time Standard: Guidance. London: NHS England Publications. 2016.

For peer review only

[29] Julious, SA. Sample size of 12 per group rule of thumb for a pilot study. Pharm Stat. 2005;287-91.

[30] Ofcom. The Communications Market Report. London, UK: Ofcom; 2017

[31] Plaistow J, Birchwood M. Back in the saddle: a guide to relapse prevention. In: Birchwood M, Fowler D, Jackson C, editors. Early Intervention in Psychosis: A Guide to Concepts, Evidence and Interventions. Chichester: McGraw-Hill; 1996. p. 239-244.

[32] Perkins R, Rinaldi M. Taking back control: a guide to planning your own recovery. South West London: St George's Mental Health NHS Trust, 2007.

[33] Tait L, Birchwood M, Trower P. A new scale (SES) to measure engagement with community mental health services. J Ment Health. 2002;11:191-8.

[34] Kay SR, Fiszbein A, Opfer LA. The positive and negative syndrome scale (PANSS) for schizophrenia. Schizophrenia Bull. 1987;13:261-76.

[35] Priebe S, Watzke S, Hansson L, et al. Objective social outcomes index (SIX): a method to summarise objective indicators of social outcomes in mental health care. Acta Psychiat Scand. 2008;118:57-63.

[36] Carpinello SE, Knight EL, Markowitz FE, et al. The development of the Mental Health Confidence Scale: A measure of self-efficacy in individuals diagnosed with mental disorders. Psychiatr Rehabil J. 2000;23:236-243.

[37] Neil ST, Kilbride M, Pitt L, et al. The questionnaire about the process of recovery (QPR): a measurement tool developed in collaboration with service users. Psychosis. 2009;1:145-55.

[38] Tennant R, Hiller L, Fishwick R, et al. The Warwick-Edinburgh mental well-being scale (WEMWBS): development and UK validation. Health Qual Life Out. 2007;5:63.

For peer review only

[39] Priebe S, McCabe RO, Bullenkamp J, et al. Structured patient–clinician communication and 1-year outcome in community mental healthcare. Brit J Psychiat. 2007;191:420-6.

[40] Sekhon M, Cartwright M, Francis JJ. Acceptability of healthcare interventions: an overview of reviews and development of a theoretical framework. BMC Health Serv Res. 2017:17:88.

For peer review only

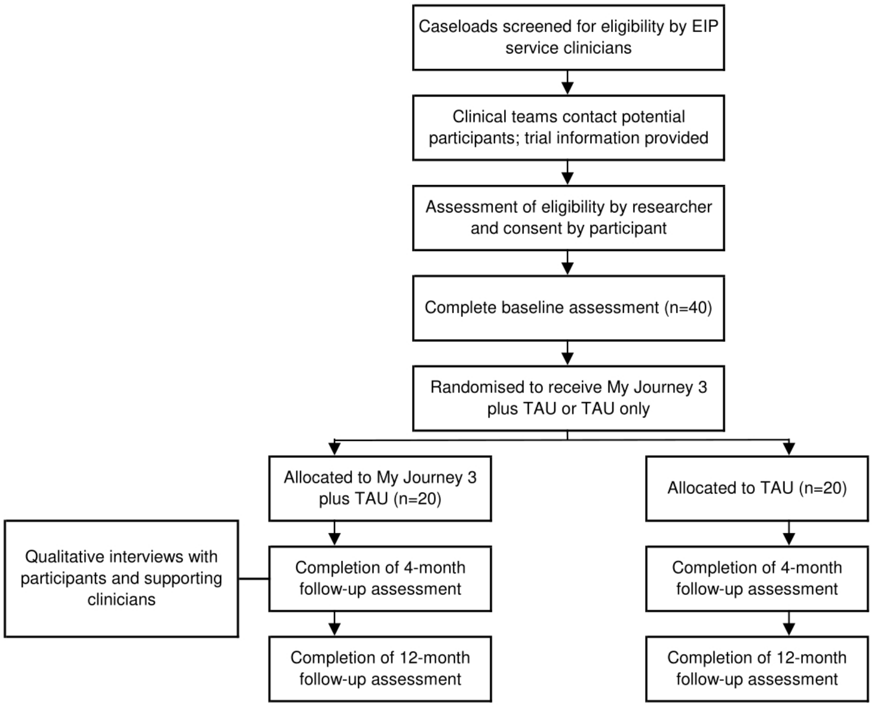

SPIRIT flow diagram outlining the phases of the ARIES feasibility trial.

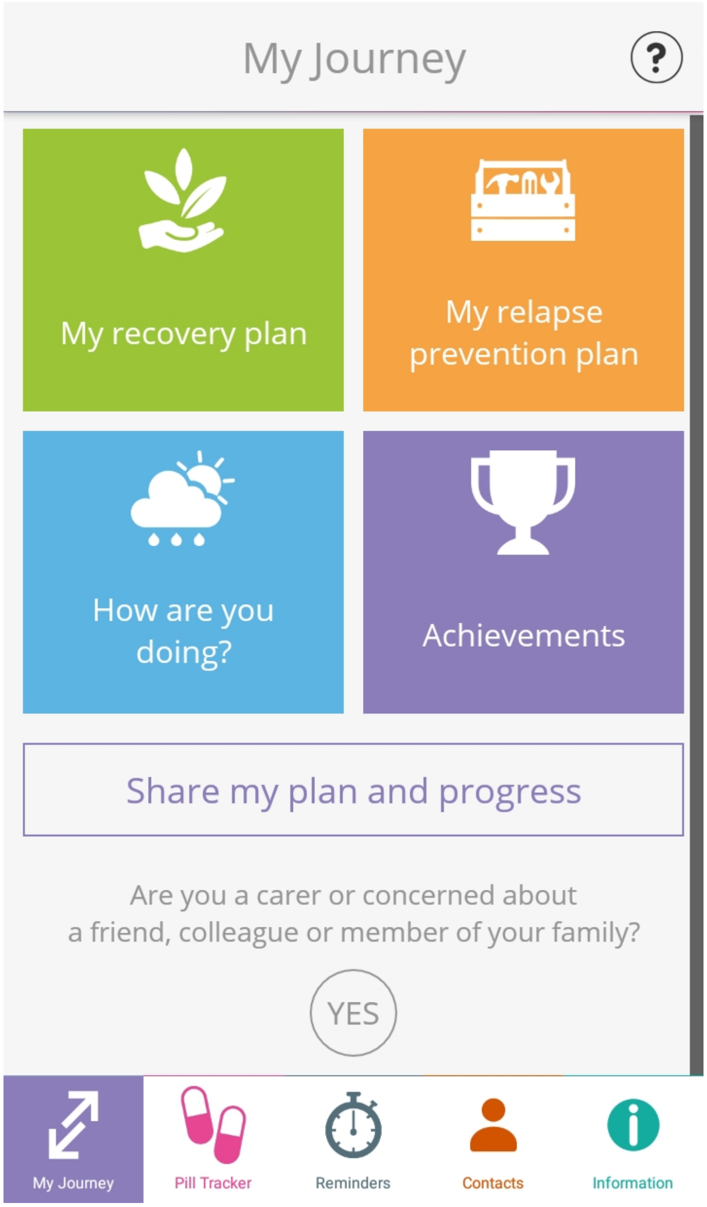

The My Journey 3 home screen, which is seen by the user when accessing the app on their Smartphone.

SPIRIT 2013 Checklist: Recommended items to address in a clinical trial protocol and related documents*

**Section/item**	**Item No**	**Description**	**Addressed on page number**
**Administrative information**			*Sections headings given until page numbers finalised*
Title	1	Descriptive title identifying the study design, population, interventions, and, if applicable, trial acronym	Title page
Trial registration	2a	Trial identifier and registry name. If not yet registered, name of intended registry	Abstract page
	2b	All items from the World Health Organization Trial Registration Data Set	Additional file 2
Protocol version	3	Date and version identifier	Ethics and dissemination - Ethical approval
Funding	4	Sources and types of financial, material, and other support	Declarations - funding
Roles and responsibilities	5a	Names, affiliations, and roles of protocol contributors	Title page/ Declarations - authors' contributions
	5b	Name and contact information for the trial sponsor	Title page

For peer review only

	5c	Role of study sponsor and funders, if any, in study design; collection, management, analysis, and interpretation of data; writing of the report; and the decision to submit the report for publication, including whether they will have ultimate authority over any of these activities	Declarations - funding
	5d	Composition, roles, and responsibilities of the coordinating centre, steering committee, endpoint adjudication committee, data management team, and other individuals or groups overseeing the trial, if applicable (see Item 21a for data monitoring committee)	Methods and analysis – Patient and Public Involvement/data monitoring and management
**Introduction**			
Background and rationale	6a	Description of research question and justification for undertaking the trial, including summary of relevant studies (published and unpublished) examining benefits and harms for each intervention	Introduction
	6b	Explanation for choice of comparators	Methods and analysis - the intervention
Objectives	7	Specific objectives or hypotheses	Introduction - aims and objectives
Trial design	8	Description of trial design including type of trial (eg, parallel group, crossover, factorial, single group), allocation ratio, and framework (eg, superiority, equivalence, noninferiority, exploratory)	Methods and analysis - design
**Methods: Participants, interventions, and outcomes**			
Study setting	9	Description of study settings (eg, community clinic, academic hospital) and list of countries where data will be collected. Reference to where list of study sites can be obtained	Methods and analysis - setting
Eligibility criteria	10	Inclusion and exclusion criteria for participants. If applicable, eligibility criteria for study centres and individuals who will perform the interventions (eg, surgeons, psychotherapists)	Methods and analysis - eligibility criteria

For peer review only

Interventions	11a	Interventions for each group with sufficient detail to allow replication, including how and when they will be administered	Methods and analysis - the intervention/ treatment as usual
	11b	Criteria for discontinuing or modifying allocated interventions for a given trial participant (eg, drug dose change in response to harms, participant request, or improving/worsening disease)	Ethics and dissemination - serious adverse events
	11c	Strategies to improve adherence to intervention protocols, and any procedures for monitoring adherence (eg, drug tablet return, laboratory tests)	Methods and analysis - the intervention
	11d	Relevant concomitant care and interventions that are permitted or prohibited during the trial	Methods and analysis - treatment as usual
Outcomes	12	Primary, secondary, and other outcomes, including the specific measurement variable (eg, systolic blood pressure), analysis metric (eg, change from baseline, final value, time to event), method of aggregation (eg, median, proportion), and time point for each outcome. Explanation of the clinical relevance of chosen efficacy and harm outcomes is strongly recommended	Methods and analysis - measures
Participant timeline	13	Time schedule of enrolment, interventions (including any run-ins and washouts), assessments, and visits for participants. A schematic diagram is highly recommended (see Figure)	Figure 1
Sample size	14	Estimated number of participants needed to achieve study objectives and how it was determined, including clinical and statistical assumptions supporting any sample size calculations	Methods and analysis - participants
Recruitment	15	Strategies for achieving adequate participant enrolment to reach target sample size	Methods and analysis - recruitment
**Methods: Assignment of interventions (for controlled trials)**			

Allocation:			
Sequence generation	16a	Method of generating the allocation sequence (eg, computer-generated random numbers), and list of any factors for stratification. To reduce predictability of a random sequence, details of any planned restriction (eg, blocking) should be provided in a separate document that is unavailable to those who enrol participants or assign interventions	Methods and analysis - randomisation
Allocation concealment mechanism	16b	Mechanism of implementing the allocation sequence (eg, central telephone; sequentially numbered, opaque, sealed envelopes), describing any steps to conceal the sequence until interventions are assigned	Methods and analysis - randomisation
Implementation	16c	Who will generate the allocation sequence, who will enrol participants, and who will assign participants to interventions	Methods and analysis - randomisation
Blinding (masking)	17a	Who will be blinded after assignment to interventions (eg, trial participants, care providers, outcome assessors, data analysts), and how	Methods and analysis - randomisation
	17b	If blinded, circumstances under which unblinding is permissible, and procedure for revealing a participant's allocated intervention during the trial	N/A
**Methods: Data collection, management, and analysis**			
Data collection methods	18a	Plans for assessment and collection of outcome, baseline, and other trial data, including any related processes to promote data quality (eg, duplicate measurements, training of assessors) and a description of study instruments (eg, questionnaires, laboratory tests) along with their reliability and validity, if known. Reference to where data collection forms can be found, if not in the protocol	Methods and analysis - data collection
	18b	Plans to promote participant retention and complete follow-up, including list of any outcome data to be collected for participants who discontinue or deviate from intervention protocols	Methods and analysis - recruitment

For peer review only - http://bmjopen.bmj.com/site/about/guidelines.xhtml

Data management	19	Plans for data entry, coding, security, and storage, including any related processes to promote data quality (eg, double data entry; range checks for data values). Reference to where details of data management procedures can be found, if not in the protocol	Methods and analysis - data monitoring and management
Statistical methods	20a	Statistical methods for analysing primary and secondary outcomes. Reference to where other details of the statistical analysis plan can be found, if not in the protocol	Methods and analysis - data analysis
	20b	Methods for any additional analyses (eg, subgroup and adjusted analyses)	N/A
	20c	Definition of analysis population relating to protocol non-adherence (eg, as randomised analysis), and any statistical methods to handle missing data (eg, multiple imputation)	Methods and analysis - data analysis
**Methods: Monitoring**			
Data monitoring	21a	Composition of data monitoring committee (DMC); summary of its role and reporting structure; statement of whether it is independent from the sponsor and competing interests; and reference to where further details about its charter can be found, if not in the protocol. Alternatively, an explanation of why a DMC is not needed	Methods and analysis - data monitoring and management
	21b	Description of any interim analyses and stopping guidelines, including who will have access to these interim results and make the final decision to terminate the trial	Methods and analysis - data analysis
Harms	22	Plans for collecting, assessing, reporting, and managing solicited and spontaneously reported adverse events and other unintended effects of trial interventions or trial conduct	Ethics and dissemination - serious adverse events
Auditing	23	Frequency and procedures for auditing trial conduct, if any, and whether the process will be independent from investigators and the sponsor	N/A
**Ethics and dissemination**			

Research ethics approval	24	Plans for seeking research ethics committee/institutional review board (REC/IRB) approval	Ethics and dissemination - ethical approval
Protocol amendments	25	Plans for communicating important protocol modifications (eg, changes to eligibility criteria, outcomes, analyses) to relevant parties (eg, investigators, REC/IRBs, trial participants, trial registries, journals, regulators)	Ethics and dissemination - ethical approval
Consent or assent	26a	Who will obtain informed consent or assent from potential trial participants or authorised surrogates, and how (see Item 32)	Methods and analysis - recruitment
	26b	Additional consent provisions for collection and use of participant data and biological specimens in ancillary studies, if applicable	N/A
Confidentiality	27	How personal information about potential and enrolled participants will be collected, shared, and maintained in order to protect confidentiality before, during, and after the trial	Ethics and dissemination - confidentiality
Declaration of interests	28	Financial and other competing interests for principal investigators for the overall trial and each study site	Declarations - competing interests
Access to data	29	Statement of who will have access to the final trial dataset, and disclosure of contractual agreements that limit such access for investigators	Methods and analysis - data monitoring and management
Ancillary and post-trial care	30	Provisions, if any, for ancillary and post-trial care, and for compensation to those who suffer harm from trial participation	N/A

For peer review

For peer review only - http://bmjopen.bmj.com/site/about/guidelines.xhtml

Dissemination policy	31a	Plans for investigators and sponsor to communicate trial results to participants, healthcare professionals, the public, and other relevant groups (eg, via publication, reporting in results databases, or other data sharing arrangements), including any publication restrictions	Ethics and dissemination – dissemination/ Methods and analysis – patient and public involvement
	31b	Authorship eligibility guidelines and any intended use of professional writers	Declarations – authors' contributions
	31c	Plans, if any, for granting public access to the full protocol, participant-level dataset, and statistical code	Data statement
**Appendices**			
Informed consent materials	32	Model consent form and other related documentation given to participants and authorised surrogates	Additional file 3
Biological specimens	33	Plans for collection, laboratory evaluation, and storage of biological specimens for genetic or molecular analysis in the current trial and for future use in ancillary studies, if applicable	N/A

*It is strongly recommended that this checklist be read in conjunction with the SPIRIT 2013 Explanation & Elaboration for important clarification on the items. Amendments to the protocol should be tracked and dated. The SPIRIT checklist is copyrighted by the SPIRIT Group under the Creative Commons "Attribution-NonCommercial-NoDerivs 3.0 Unported" license.

For peer review only - http://bmjopen.bmj.com/site/about/guidelines.xhtml

World Health Organization Trial Registration Data Set

Primary Registry and Trial Identifying Number	ISRCTN10004994
Date of Registration in Primary Registry	25/05/2018
Secondary Identifying Numbers	IRAS ID: 182553<br>REC reference: 15/LO/1453
Source(s) of Monetary or Material Support	The research is funded by the National Institute for Health Research (NIHR) Collaboration for Leadership in Applied Health Research and Care North Thames at Barts Health NHS Trust (NIHR CLAHRC North Thames).
Primary Sponsor	Camden and Islington NHS Foundation Trust
Secondary Sponsor(s)	N/A
Contact for Public Queries	Thomas Steare<br><br>+44 (0)20 7679 8192<br>thomas.steare.15@ucl.ac.uk<br>UCL Division of Psychiatry<br>6th Floor, Maple House<br>149 Tottenham Court Road<br>London<br>W1T 7NF
Contact for Scientific Queries	Prof Sonia Johnson<br><br>+44 (0)20 7679 9453<br>s.johnson@ucl.ac.uk<br>UCL Division of Psychiatry<br>6th Floor, Maple House<br>149 Tottenham Court Road<br>London<br>W1T 7NF
Public Title	ARIES: App to support Recovery in Early Intervention Services
Scientific Title	App to support Recovery In Early intervention Services (the ARIES study): feasibility trial of a supported self-management Smartphone application for psychosis
Countries of Recruitment	England
Health Condition(s) or Problem(s) Studied	First-episode psychosis
Intervention(s)	Self-management Smartphone app (My Journey 3)
Key Inclusion and Exclusion Criteria	Participant inclusion criteria

For peer review only

	1. Currently on the caseload of an Early Intervention Service and in contact with clinicians<br>2. Aged 16 or older<br>3. Have a diagnosis of psychosis<br>4. Own an Android Smartphone.<br><br>Participant exclusion criteria<br><br>1. Lack capacity to provide consent to take part in the study<br>2. Unable to communicate and understand English sufficiently to understand trial procedures and use the app<br>3. In the view of their EIS team, pose such a high risk to others that it would be unsafe to conduct research meetings even on NHS premises.
Study Type	Multi-centre randomised controlled feasibility trial
Date of First Enrolment	09/03/2017
Sample Size	40
Recruitment Status	Recruitment target met – no longer recruiting
Primary Outcome(s)	Relapse as indicated by admission to acute care (inpatient wards, crisis resolution teams, crisis houses and acute day services) during the 12-month follow-up period. Data on admissions to acute care during the trial period is collected from patient records at the 12-month follow-up.
Key Secondary Outcomes	1. Social outcomes are measured using The Social Outcomes Index (Priebe, Watzke, Hansson & Burns, 2008) at the study baseline meeting, at a 4-month follow-up meeting and at a 12-month follow-up meeting<br>2. Mental wellbeing is assessed using The Mental Health Confidence Scale (Carpinello et al., 2000) and The Warwick-Edinburgh Mental Well-Being Scale (NHS Health Scotland, University of Warwick & University of Edinburgh, 2007) at the study baseline meeting, at a 4-month follow-up meeting and at a 12-month follow-up meeting<br>3. Recovery in psychosis is assessed using The Process of Recovery Questionnaire (Neil et al., 2009) at the study baseline meeting, at a 4-month follow-up meeting and at a 12-month follow-up meeting<br>4. Quality of life and satisfaction with treatment is assessed using The DIALOG scale (Priebe et al., 2007) at the study baseline meeting, at a 4-month follow-up meeting and at a 12-month follow-up meeting<br>5. Positive, negative and general psychopathology symptoms are assessed using the PANSS (Kat et al., 1987) at the study

For peer review only

	baseline meeting, at a 4-month follow-up meeting and at a 12-month follow-up meeting<br>6. Participants' engagement with Early Intervention Services during the study period is obtained using the Service Engagement Scale (SES; Tait et al., 2002) completed by participants' clinicians at baseline and at the 12-month follow-up.<br>7. The following patient information is collected from patient records at baseline and one year after entry into the study:<br>7.1. Current diagnosis<br>7.2. Current care cluster<br>7.3. Care plan approach status<br>8. The usability and acceptability of My Journey 3 for service users and clinicians is assessed from semi-structured qualitative interviews conducted at the 4-month follow-up meeting.
Ethics Review	National Research Ethics Service Committee London - Brent, 02/10/2015, ref: 15/LO/1453. Amendment approved 29/07/2017.
Completion date	Study is ongoing

For peer review only

Service user participant consent form

Study Title: App to support Recovery In Early intervention Services (the ARIES study): Pilot randomised controlled trial of a self-management smartphone application

Principal Investigators: Professor Sonia Johnson and Professor David Osborn

Please Initial Each Box

1. I confirm that I have read and understood the Participant Information Sheet V5 dated 29/05/2017 for the above study and have had the opportunity to ask questions about the study. ☐

2. I understand that my participation is voluntary and that I am free to withhold personal information or to withdraw my participation at any time, without giving any reason, and without my medical care or legal rights being affected. ☐

3. I understand that if I choose to withdraw from the study that any data that I have already provided for the purposes of the research will be kept and used by the research team. ☐

4. I give permission for my General Practitioner (GP) and my Early Intervention team to be told I am participating in this study. ☐

5. I understand that relevant sections of my medical notes and data collected during the study may be looked at by regulatory authorities or from the NHS Trust, where it is relevant to my taking part in this research. I give permission for these individuals to have access to my records. ☐

6. I understand that I will be given a £20 gift as cash for my participation in each study assessment. ☐

7. I agree to the research team consulting NHS electronic records to investigate my diagnosis, medication, and mental health service use, and give them permission to do so even if I choose to no longer participate in the intervention, or they are not able to carry out further study interviews with me. ☐

8. I understand that in the event that I disclose information which may indicate new risk to myself or others, the researcher will be obliged to follow NHS Trust risk procedures that may require release of my personal data. ☐

9. I give permission for findings from the study to be written up for publication. Any publication will not identify me. ☐

10. I give permission to be audio recorded where required for the purposes of the study. I understand these audio-recordings will be transcribed and anonymised and audio recordings destroyed after the study. I give permission for direct quotations taken from this interview to be included in papers written for publication. Any quotation would not identify me. ☐

11. I give permission for the research team to collect data from the My Journey 3 app regarding the frequency, duration, and pattern of my use of it. I understand that no personal information will be collected from the app. ☐

12. I give permission for non-identifiable data to be shared with other research teams for research purposes. ☐

For peer review only - http://bmjopen.bmj.com/site/about/guidelines.xhtml

13. I agree to take part in this study. ☐

_________________________ _______________ _________________________

Name of participant Date Signature

_________________________ _______________ ________________________

Name of Researcher taking consent Date Signature

For peer review only

For peer review only - http://bmjopen.bmj.com/site/about/guidelines.xhtml

BMJ Open

The App to support Recovery in Early Intervention Services (ARIES) Study: Protocol of a feasibility randomised controlled trial of a self-management Smartphone application for psychosis

Journal:	*BMJ Open*
Manuscript ID	bmjopen-2018-025823.R2
Article Type:	Protocol
Date Submitted by the Author:	04-Jan-2019

Complete List of Authors:	Steare, Thomas; University College London, Division of Psychiatry; Camden and Islington NHS Foundation Trust,<br>O'Hanlon, Puffin; University College London, Division of Psychiatry<br>Eskinazi, Michelle; University College London, Division of Psychiatry<br>Osborn, David; UCL, Faculty of Brain Sciences; Camden and Islington NHS Foundation Trust<br>Lloyd-Evans, Brynmor ; UCL, Division of Psychiatry; Camden and Islington NHS Foundation Trust<br>Jones, Rebecca; UCL, Division of Psychiatry<br>Rostill, Helen; Surrey and Borders Partnership NHS Foundation Trust<br>Amani, Sarah; NHS England, Early Intervention in Psychosis Programme (South of England)<br>Johnson, Sonia; UCL, Division of Psychiatry ; Camden and Islington NHS Foundation Trust

**Primary Subject Heading**:	Mental health
Secondary Subject Heading:	Mental health
Keywords:	Schizophrenia & psychotic disorders < PSYCHIATRY, MENTAL HEALTH, PSYCHIATRY

SCHOLARONE™ Manuscripts

TITLE PAGE

Title: The App to support Recovery in Early Intervention Services (ARIES) Study: Protocol of a feasibility randomised controlled trial of a self-management Smartphone application for psychosis

Authors:

Thomas Steare

Division of Psychiatry, University College London, London, UK

R&D Department, Camden and Islington NHS Foundation Trust, London, UK

Puffin O'Hanlon

Division of Psychiatry, University College London, London, UK

Michelle Eskinazi

Division of Psychiatry, University College London, London, UK

David Osborn

Division of Psychiatry, University College London, London, UK

R&D Department, Camden and Islington NHS Foundation Trust, London, UK

Brynmor Lloyd-Evans

Division of Psychiatry, University College London, London, UK

R&D Department, Camden and Islington NHS Foundation Trust, London, UK

Rebecca Jones

Division of Psychiatry, University College London, London, UK

Helen Rostill

Surrey and Borders Partnership NHS Foundation Trust, Leatherhead, Surrey, UK

For peer review only

Sarah Amani

Senior Programme Manager, EIP Programme (South of England), NHS England, Oxford, Oxfordshire, UK

Sonia Johnson (Corresponding author)

Address: UCL Division of Psychiatry, 6th Floor, Wing B, Maple House, 149 Tottenham Court Road, London W1T 7NF

Division of Psychiatry, University College London, London, UK

R&D Department, Camden and Islington NHS Foundation Trust, London, UK

s.johnson@ucl.ac.uk

02076799453

Trial Sponsor: Camden & Islington NHS Foundation Trust. sponsor.noclor@nhs.net

Study start date (first participant recruited): 09 March 2017

Study end date: 31 March 2019

Keywords: Psychosis, Self-management, Smartphone, Feasibility study, Randomised controlled trial

Word count: 4,756

For peer review only

ABSTRACT

Introduction: Mental health interventions delivered through digital technology have potential applications in promoting recovery and improving outcomes among people in the early stages of psychosis. Self-management approaches are recommended for the treatment of psychosis and could be delivered via applications (apps) installed on Smartphones to provide low-cost accessible support. We describe the protocol for a feasibility trial investigating a self-management Smartphone app intervention for adults using Early Intervention in Psychosis (EIP) services.

Methods and analysis: In this feasibility randomised controlled trial, 40 participants will be recruited from EIP services in London and Surrey. Twenty participants will be randomised to receive a supported self-management Smartphone app (My Journey 3) plus Treatment As Usual (TAU), while the other 20 participants will receive TAU only. The primary objective of this study is to evaluate the feasibility of conducting a full-scale trial of this intervention in EIP services. Participant data will be collected at baseline and at two follow-up assessments conducted four months and 12 months post-baseline. Analysed outcome measures will include relapse of psychosis (operationalised as admission to a hospital or community acute alternative), mental health and wellbeing, recovery, quality of life and psychopathology. Semi-structured interviews with participants and EIP service clinicians will additionally explore experiences of using My Journey 3 and participating in the trial and suggestions for improving the intervention.

Ethics and dissemination: The ARIES study has been reviewed and approved by the National Research Ethics Service Committee London – Brent (Research Ethics Committee reference: 15/LO/1453). The findings of this study will be disseminated through peer-reviewed scientific journals and conferences, magazines and web publications.

For peer review only

Trial registration number: ISRCTN10004994

ARTICLE SUMMARY

Strengths and limitations of this study

- The app has been well developed prior to the feasibility trial, including substantial stakeholder input to the development of the intervention at all stages, an initial period of testing in clinical settings, and refinement of the current version through lab and field testing.
- The acceptability and feasibility of the intervention and the study design of a future full-scale trial will be explored using a variety of measures, including recruitment and retention rates, usage data, and qualitative interviews on experience of use.
- The trial will only recruit Android Smartphone users as My Journey 3 is not as yet compatible with iOS or other Smartphone operating systems; this will limit sample representativeness.
- This is a feasibility study, thus not powered to draw conclusions regarding the effectiveness of the intervention.

For peer review only

INTRODUCTION

Psychosis is associated with significant costs at individual-, family- and society-level.[1-3] The implementation of Early Intervention in Psychosis (EIP) services across the United Kingdom has been associated with improved outcomes,[4,5] however substantial challenges remain. Long-term follow-up studies have shown that relapse rates in the early course of psychosis remain high.[6] Furthermore functional recovery is often not attained following treatment.[7]

Thus there is a need to increase and sustain benefits from EIP services. An approach with demonstrated efficacy in improving functional recovery and reducing relapse in psychosis is supported self-management.[8] Self-management has been designed to empower people as active agents in their own recovery by enabling them to develop skills such as recognising and monitoring symptoms and early warning signs of relapse, identifying and avoiding stressors, and using effective coping strategies.[9] Self-management is associated with significant benefits including reduced distress, improved medication adherence and reduced number of hospitalizations.[10-12] Relapse prevention work has long been proposed as a key element in EIP,[13] and establishing and working towards recovery goals is widely advocated in EIP services.[14] However, well-evaluated tools and methods are currently not available to support widespread implementation of self-management approaches in EIP services.

Over recent years there has been growing interest in the development of innovative technologies in health-care due to their potential to improve accessibility, efficacy, quality and cost-effectiveness of treatment.[15] Due to rapid advancements in mobile phone technology, it is possible to deliver clinical interventions via applications (apps) installed on Smartphones (mobile phones with computational capacities). Smartphones are widely available in the UK with over 75% of adults owning one.[16] They are often carried on the person with high

For peer review only

accessibility to the internet and are therefore a suitable device to provide time-unlimited interventions in almost any location.[17]

Recent evidence suggests that people with psychosis are adopting digital technology in a similar way to the general population,[18-20] and that they are interested in using mental health interventions delivered via Smartphones.[21,22] A systematic review has found that interventions delivered through apps or text messaging on Smartphones are acceptable and feasible for people with psychosis and may support recovery.[23] Smartphone apps based on self-management principles have shown promise in a wide range of long-term health conditions,[24] and are a potential way to deliver accessible low-cost support to adults with psychosis.

Although emerging research suggests that Smartphone apps hold promise in delivering effective interventions to adults with psychosis, the evidence base is under-developed in comparison to more common mental disorders.[25] To date only one randomised controlled trial (RCT) of a psychosocial Smartphone app used in EIP services has been published.[26] In this proof-of-concept trial, 36 adults accessing EIP services were randomised to use a cognitive-behavioural therapy informed Smartphone app (Actissist) that aims to encourage active self-management, or a symptom-monitoring app which was classed as the control condition. Actissist was found to be acceptable, feasible and safe for adults accessing EIP services. Although not powered to find an effect, the study suggests that Actissist may confer benefits to users' outcomes over and above a passive symptom-monitoring app. Participants however only had access to the app for 12 weeks, were followed up over a relatively short timeframe (22 weeks) and significant mental health outcomes such as relapse were not measured. This work has been conducted in parallel with our study, and we anticipate both programmes of work will contribute to developing an evidence base as to whether Smartphone

For peer review only

apps that promote self-management can improve outcomes in first-episode psychosis and reduce healthcare costs.

Aims and objectives

The aim of the ARIES study is to examine the feasibility of conducting a full-scale trial of a clinician supported self-management Smartphone app for adults accessing EIP services. The feasibility trial will aim:

1. To identify whether a self-management Smartphone app is acceptable to use and feasible to support in EIP services in the context of a research study, and to identify any necessary modifications to the intervention content and design, or to its delivery in EIP services.
2. To test the feasibility and acceptability of trial procedures for a definitive trial.
3. To test procedures for evaluating intervention engagement and participant outcomes.

METHODS AND ANALYSIS

Design

The study is a feasibility RCT comparing a clinician supported self-management Smartphone app (My Journey 3), in addition to Treatment As Usual (TAU), to a control group receiving TAU only. The design as described here adheres to the Standard Protocol Items: Recommendations for Interventional Trials (SPIRIT).[27] A copy of the SPIRIT checklist is provided as Additional file 1. Relevant items from the World Health Organization Trial Registration Data Set are detailed in Additional file 2.

Setting

The trial will take place in six EIP services across three NHS Foundation Trusts in England; Camden and Islington, East London, and Surrey and Borders Partnership. All

For peer review only

participating EIP services provide care coordination to service users, access to a psychiatrist and psychiatric medication and psychosocial interventions, aiming to conform to the current UK model for EIP services.[28] None of the participating EIP services offer Smartphone apps or any other digital interventions as part of routine care, nor are structured self-management tools consistently available in these teams. A list of participating sites is available from the authors.

Participants

Forty service users will be recruited from the participating EIP services. Assuming a conservative estimate of a 40% attrition rate, the study should feature 12 completer participants in each group as recommended for feasibility and pilot trials.[29] The proposed sample size will therefore be sufficient to establish the acceptability of My Journey 3 and the feasibility of trial procedures.

Eligibility criteria

Inclusion criteria

- Aged 16 or older
- Have experienced at least one episode of psychosis
- Currently on the caseload of an EIP service and in contact with clinicians
- User of a Smartphone with an Android operating system.

Exclusion criteria

- Lack of capacity to provide informed consent to participate in the trial
- Inability to communicate and understand English sufficiently to understand trial procedures and use My Journey 3

For peer review only

- In the view of their EIP service, poses such a high risk to others that it would be unsafe to conduct research meetings even on NHS premises.

Criteria are deliberately broad in order to reach conclusions generalizable to a wide range of EIP service users. Due to limited resources My Journey 3 has been developed for one Smartphone operating system (Android) at this stage of testing. Nearly half of Smartphones used in the UK utilise Android operating systems.[30] There is also emerging evidence that Android may be the Smartphone operating system most often used by adults with severe mental illness.[21] Resources are not currently available to deliver My Journey 3 in languages other than English.

Recruitment

An overview of the recruitment procedure is displayed in the flow diagram (figure 1). Clinicians will identify EIP service users who appear potentially eligible for the trial. They will then make initial contact, explain the trial and ask if the potential participant is willing to be contacted by a researcher. If a potential participant is eligible and interested in taking part, a researcher will contact them to further explain the trial and to arrange a face-to-face meeting. At this meeting the researcher will provide the trial information sheet, confirm eligibility, invite any questions about participating and assess the service user's capacity to provide informed consent. Written consent to participate will be obtained using a consent form (Additional file 3) prior to undertaking the baseline assessment at the same meeting. Participants will be notified that they will be free to withdraw from the study at any time-point. Prior to study recruitment, researchers will undertake training in how to assess mental capacity and take informed consent.

For peer review only

To minimize loss to follow-up, participants will be asked to give their preferred contact details, and for their permission for the researcher to contact a nominated close other or EIP service clinician whom could contact the participant if the researcher is unable to contact the participant directly.

Randomisation

At a separate time-point following the baseline assessment meeting participants will be randomly allocated in a 1:1 ratio to an intervention group where they will have access to My Journey 3 during the trial (n=20) or to a control group (n=20). Randomisation will be conducted by an independent statistician. The allocation sequence will be concealed from the researcher, who will be blind when recruiting participants and conducting baseline assessments. An independent researcher will hold on to the allocation list and disclose participants' allocation to the trial researcher after the completion of the baseline assessments. Participants will then be informed of their allocation by the trial researcher. Participants cannot be blinded to their group allocation given the nature of the trial intervention and control group. As a single researcher will carry out most of the data collection, it is not practical for the group allocation of participants to be concealed from the research team in this feasibility study.

For peer review only

The intervention

Development

My Journey 3 has been developed through a series of iterations. The first version of My Journey was designed by Surrey and Borders Partnership NHS Foundation Trust (led by Sarah Amani), after local EIP service users consulted about their care suggested there would be significant benefits from a Smartphone app that could be used for appointment and medication reminders, to track their mood and to share their recovery progress with EIP staff or carers. In

2011 a project group consisting of EIP service users, NHS clinicians, a pharmacist and a NHS manager was formed to drive the development of the My Journey app. The following year a prototype of My Journey was tested by 20 EIP service users to further inform development of the app. Since April 2013 this version of My Journey has been available for download from Google Play (the official app store for Android Smartphones).

The original version of My Journey contains generic advice on what to do if certain clinical difficulties arise, but does not allow personalised relapse prevention planning or recovery goal tracking. User feedback suggested that this was a limitation. In the current study we have collaborated with digital health experts, EIP service clinicians and adults with lived experience of psychosis to adapt existing paper-and-pen self-management intervention components – in routine use in NHS services - to be suitable for delivery in an app format .[31,32] In creating the product specification of My Journey 3 we have incorporated these self-management components with functions from the original My Journey. Technical development of My Journey 3 has been led by MyOxygen (https://myoxygen.uk/), a private app development company based in the UK.

Following the initial development of My Journey 3, it has been refined in response to two phases of preliminary testing. In the first phase six volunteer EIP services users participated in usability lab tests with My Journey 3 on their own Android Smartphone. Usability testing featured “think aloud” tests where participants completed set tasks using My Journey 3 while providing a continuous commentary on their thoughts. This method was used to highlight design and usability issues and users’ immediate reactions to the app. Individual interviews were also conducted to explore participants’ perceptions of the ease of use of My Journey 3, concerns they might have and suggestions for improvements. My Journey 3 was modified to reflect the findings at this stage.

For peer review only

In the second phase a further six volunteer service users trialled My Journey 3 on their own Android Smartphone during a one-month field study, with EIP service clinicians asked to support their clients' use of the app during routine appointments. Following the field study, individual interviews were conducted with service user participants to explore how they used My Journey 3 and how it could be improved. Further interviews with EIP service clinicians explored their experience of supporting their client with My Journey 3. Based on these findings My Journey 3 was updated again prior to the feasibility trial.

Intervention outline

My Journey 3 is a Smartphone app that has been designed to be used alongside EIP service care and with the support of clinicians. My Journey 3 aims to develop and support users' illness self-management skills to help facilitate recovery from first-episode psychosis. The My Journey 3 home screen can be seen in figure 2.

Three main components of My Journey 3 have been directly taken from the original app and updated. Information regarding psychosis, mental health and mental health services is provided in My Journey 3 through links to relevant NHS and voluntary sector websites and through videos of personal recovery stories. To facilitate symptom recognition and monitoring My Journey 3 also features a self-monitoring tool and symptom tracker where users can monitor 14 different symptoms and 3 lifestyle behaviours. Advice to help manage symptoms is provided after completing the symptom tracker, and a graphical summary of symptom severity over time is displayed. Users also have access to a pill tracker where they can log whether they have taken their psychiatric medication. The pill tracker features a daily alert at a pre-set time to remind users to input if they have taken their medication.

For peer review only

In designing My Journey 3 we have also drawn on evidenced-based self-management interventions to add structured intervention components focused on recovery planning and relapse prevention.[31,32] These additions allow for users to interactively:

- Identify strategies and coping resources that they find useful in maintaining wellbeing
- Set and track progress towards personal recovery goals
- Identify personal early warning signs of relapse and strategies and coping mechanisms to put in place should they experience these
- Create a "relapse plan", an action plan to follow in times of crisis in order to avoid or attenuate relapse

My Journey 3 has been designed to be used by EIP service users in collaboration with clinicians. Clinicians can input relevant information to sections such as the relapse prevention plans and, with training, can provide assistance with the app. It is also suitable for independent use: the developers' aspiration is for the app to be initially used in collaboration with clinicians, but for it then to support users' self-management following discharge from EIP services.

My Journey 3 features weekly discrete notifications that appear on the users' Smartphone interface to encourage engagement with the app. Users also have the opportunity to set activity reminders that notify the user to engage in coping strategies and take part in pre-identified activities to promote wellbeing.

My Journey 3 also features a sharing functionality for users who wish to share their data with clinicians, family members, friends or other trusted third parties. This uses built-in sharing functionality of the user's Smartphone, such as e-mail. Participants will have control of who they choose to share their data with at all times.

Delivery of the intervention

For peer review only

Each participant in the intervention group will take part in an individual training session with their supporting EIP service clinician and a researcher within six weeks of the initial consent meeting. During each training session participants will download My Journey 3 on to their Smartphone. The researcher will then give a demonstration of the app and its main functions. Participants will have the opportunity to practice using My Journey 3 and to ask questions. To facilitate use during the trial participants will be asked to input appropriate information in to the main functions of My Journey 3..

Participants will have access to My Journey 3 from the training session till the 12-month time-point. Participants in the intervention group will be free to withdraw from using My Journey 3, or decline the installation of it on to their Smartphone, without any impact on their study participation or their clinical care.

In line with evidence that clinician involvement increases user engagement with Smartphone apps,[33] participants' main contact for help with using My Journey 3 and the various intervention components will be their supporting EIP service clinician. Supporting EIP service clinicians will be encouraged by the researcher to discuss recovery goals and relapse prevention plans with study participants in routine appointments and then to assist participants in inputting them into My Journey 3. Clinicians will also be encouraged to regularly check with participants if they have been using My Journey 3 and if they need any further support with it. Clinician support will not be manualised. If supporting clinicians leave the participants' EIP service the researcher will arrange a meeting to introduce any new clinicians that have clients using My Journey 3, to the intervention and how they can support service users that have access to the app. Researcher support with My Journey 3 will be limited to the initial installation and technical support as needed during the trial period.

For peer review only

The research team will have no responsibility for providing clinical care if any My Journey 3 data shared by a participant to a third party indicates a decline in their mental health. If any major concerns regarding participants' well-being such as suicidality arise from My Journey 3 data shared by a participant to the researcher, this would be communicated to the EIP service or other appropriate mental health services. Third parties such as EIP service clinicians and carers will not be briefed by the research team on how to respond to such information but would be expected to act as appropriate in such event. Participants will be informed at the training session that any data that may suggest a decline in their mental state that they have shared with EIP service clinicians or carers may be acted upon accordingly, but that My Journey 3 is not suitable for seeking urgent medical care whilst in crisis.

Control group

Participants in the control group (n=20) will receive TAU that will be unaffected by their participation.

Treatment As Usual

TAU for service users attending EIP services typically involves regular meeting with a care co-ordinator, support from multi-disciplinary clinicians and access to a psychiatrist, psychiatric medication, and a range of psychological interventions.

Data collection

The participant timeline is summarised in table 1. All participants will be asked to complete self-report questionnaires during a structured assessment with a researcher at three time points; baseline (prior to randomisation), 4-months post baseline and 12-months post baseline. Participants in the intervention group will also be invited to complete an audio-recorded interview with a researcher during the 4-month follow-up assessments. Before arranging each assessment the researcher will check with any clinicians in contact with the

For peer review only

service users that the participant does not pose a risk to others and himself/herself and that it would be safe to conduct a research meeting. If there is an identified risk too serious for a meeting on NHS premises the participant will not be met for the assessment at that time-point, with the reason for not arranging a meeting documented. If any major concerns regarding participants' risk or well-being arise during interactions with the researcher, this would be communicated to the appropriate EIP service.

Prior to the completion of each assessment participants will be provided with a trial information sheet. Their capacity to give informed consent will be assessed and their consent documented in writing. Participants will receive 20 pounds as a token of thanks for completing each assessment. Participants that have been discharged from EIP services during the trial will still be invited to attend assessments.

Once the recruitment target for the study has been met, the researcher will contact the appropriate administrators or informatics team within each NHS trust to arrange the collection of participant data from patient records. The researcher will also arrange for data to be collected from patient records one year later.

To test procedures for evaluating engagement with the intervention, data regarding My Journey 3 use will be collected throughout the trial period for all participants in the intervention group. My Journey 3 will automatically upload encrypted usage data to a secure trial server when the user has internet access on their Smartphone.

For peer review only

Table 1. Timeline of participant enrolment, interventions, assessments and patient records data collection

**Timepoint**	**Enrolment**	**Baseline**	**Allocation**	**MJ3 training session***	**4 month follow-up**	**12 month follow-up**
**Enrolment:**						
Eligibility screen	X					
Informed consent		X				
Randomisation			X			
**Intervention:**						
My Journey 3 (intervention group)				←	—	→
TAU (all participants)			←	—	—	→
**Assessments:**						
PANSS		X			X	X
Sociodemographic information		X			X	X
Clinical service use		X			X	X
Social Outcomes Index		X			X	X
Mental Health Confidence Scale		X			X	X
The Process of Recovery Questionnaire		X			X	X
WEMWBS		X			X	X
The DIALOG Scale		X			X	X
Service Engagement Scale (completed by EIP service clinicians)		X				X
Qualitative interviews (with participants in the intervention group and supporting clinicians)					X	
**Patient records data (from previous 12 months to time point):**						
Number of admissions to acute mental health services		X				X
Number of compulsory admissions to acute mental health services		X				X
Total number of days in acute care		X				X
Number of kept appointments with community mental health services		X				X
Number of missed appointments with community mental health services		X				X
Primary ICD-10 diagnosis		X				X
Most recent care cluster		X				X
Care Programme Approach status		X				X

MJ3 My Journey 3, *WEMWBS* Warwick-Edinburgh Mental Well-being Scale, *PANSS* Positive and Negative Syndrome Scale
*Participants in the intervention group only.

Measures

Data from patient records

The following data will be collected from patient records at baseline and the 12 month time-point:

a. Most recent clinical diagnosis as recorded in patient records using the ICD-10 (Internal Classification of Diseases: 10th Revision) classification.
b. Most recent care cluster (a classification of mental health service users based on their needs, used in the NHS).
c. Whether participants are subject to a care programme approach (NHS mental health services' case management model).
d. Service use during the previous 12 months measured at baseline and 12-months post study entry. Data will include history of use of acute mental health services, inpatient admissions and compulsory admissions and the number of kept and missed appointments with mental health services. Relapse of psychosis, the proposed primary outcome for a fully powered RCT, will be operationalised as participant admission to an acute mental health service (inpatient psychiatric wards, crisis houses, crisis resolution teams and acute day care services). This definition of relapse has been used previously in a recent trial of a self-management intervention in a mental health setting.[34]

My Journey 3 usage data

The data collected will be a record of each time the user opens My Journey 3, whether this was in response to a prompt, and which components they use. Usage data will not include participants' text input or responses to self-rated questions, and will therefore not give any

For peer review only

information regarding the mental health of users. Usage data will be solely used to assess the acceptability of My Journey 3 and user engagement with it.

Self-report questionnaires

The following are measured as potential secondary outcomes for a future fully-powered RCT;

Service use measures over 1 year of follow-up

1. Engagement with EIP services as measured by the Service Engagement Scale (SES),[35] a 14-item questionnaire that measures engagement for four different dimensions; availability, collaboration, help-seeking and treatment adherence. The SES will be completed by EIP service clinicians, such as participants' care co-ordinators, at baseline and at the 12-month time-point.

Measures at baseline, 4-month and 12-month follow-up assessments

1. Psychotic symptoms and general psychopathology, measured by the Positive and Negative Syndrome Scale (PANSS), a 30-item scale that yields three separate scores on positive symptoms, negative symptoms and general psychopathology.[36] To inform the PANSS a trained researcher will conduct clinical interviews with participants at each assessment.
2. Social outcomes, rated by the Social Outcomes Index (SIX),[37] a 6-item index of social outcomes and circumstance.
3. Mental health-related self-efficacy, measured by the Mental Health Confidence Scale (MHCS),[38] a 16-item self-report scale of service users' confidence in their ability to cope with stressful or difficult events.

For peer review only

4. Self-rated recovery measured by the Questionnaire about the Process of Recovery (QPR),[39] a 22-item measure, yielding a total score and subscale scores for intrapersonal and interpersonal recovery factors.
5. Mental wellbeing, rated by total score on The Warwick-Edinburgh Mental Well-Being Scale (WEMWBS),[40] a 14-item self-report scale of mental wellbeing.
6. Subjective quality of life and satisfaction with treatment measured by The DIALOG scale,[41] an 11-item self-report scale.
7. Sociodemographic characteristics including age, gender and ethnicity. We will also collect data regarding accommodation and living situation, employment status, educational attainment and Smartphone use, including use of other mental health apps.

Qualitative interviews

Interviews with participants in the intervention group will follow a topic guide and will explore their experience of using My Journey 3, including:

- The usability and acceptability of My Journey 3
- Positives and negative aspects of My Journey 3
- Impact of My Journey 3 on their life
- Facilitators and barriers to using My Journey 3
- Views on the training session
- Views on the support they received from their clinician in using My Journey 3

EIP service clinicians who have been supporting participants with My Journey 3 will also be asked to complete an audio-recorded interview close to the time the participant completes the four-month follow-up assessment. Written consent to take part will be confirmed beforehand, with an information sheet provided. Clinicians will be asked to provide demographic data prior to undertaking the interview. The interview will follow a topic guide and will focus on:

For peer review only

- Positives and negative aspects of My Journey 3
- The experience of supporting clients with My Journey 3
- Facilitators and barriers to providing support and to the incorporation of My Journey 3 in clinical management
- Views on the training session

Data analysis

No pre-specified criteria have been set for establishing the acceptability of My Journey 3 or the feasibility of trial procedures. The acceptability of My Journey 3 for EIP service users and clinicians will be determined from feedback given from the qualitative interviews and the level of participants' My Journey 3 use indicated from the app usage data. Trial feasibility will be assessed from reviewing recruitment rates, drop-out rates and intervention enrolment and use during the trial period. These will be reviewed by the study team and discussed with stakeholders at a final dissemination event to decide on future steps to take.

Quantitative analysis

We will report rates of recruitment and retention in the trial, and, for the intervention group, the level of usage of My Journey 3 during the trial. The demographic and clinical characteristics of participants at baseline will be summarised separately for each study group using descriptive statistics.

To pilot the methods of analysis for a full-scale trial we will use an intention-to-treat approach using data from all randomised participants. The effect of My Journey 3 on relapse (the proposed primary outcome for a fully-powered RCT) during the 12 month trial period will be estimated using logistic regression. The effect of the intervention on continuous outcome measures (SIX, QPR, WEMWBS, DIALOG scale, PANSS and SES) will be estimated using linear regression adjusting for the baseline measure of the outcome in question. Results will be

For peer review only

summarised using effect estimates and 95% confidence intervals only. No interim analyses are planned.

Qualitative analysis

To assess the acceptability of My Journey 3 interview data with participants in the intervention group and supporting EIP service clinicians will be analysed based on The Theoretical Framework of Acceptability for health care system interventions.[42] Analyses will be conducted collaboratively by a group of researchers.

Patient and Public Involvement

Independent advice will be sought from a trial steering group. Members will include researchers with expertise in developing and testing digital health interventions, EIP service clinicians, people with lived experience of first-episode psychosis and carers. Steering group members with lived experience of psychosis were consulted on the first paper prototypes of My Journey 3 and on the research protocol. The version of My Journey 3 tested in the current feasibility trial has been developed based on EIP service users' feedback from the usability tests and field study. Participants will be offered a summary of the research findings at the end of the study.

Data monitoring and management

A secure password-protected trial database will be developed and managed to store all quantitative data using SPSS software V.23, and will feature non-identifiable trial IDs only. Data entry of participants' questionnaires will be primarily undertaken by the trial researcher, with a random sample checked by other research team members. Anonymised electronic interview transcripts will be checked for accuracy and stored using NVivo for Windows (QSR International Pty Ltd V.11, 2016). After the trial, all data will be archived securely at University College London.

For peer review only

Due to the small sample size of the study a data monitoring committee is not planned, but the steering group will advise if one is later needed.

ETHICS AND DISSEMINATION

Ethical approval

Ethical approval has been obtained from the London Brent National Research Ethics Service Committee (Research Ethics Committee reference: 15/LO/1453), which has approved all amendments to protocol. Future protocol modifications will be submitted for approval to the research ethics committee and communicated to the study sponsor, site principle investigators, participating NHS trusts and participants. The current protocol in use is V.9, 29 July 2017.

Confidentiality

Participant data will be accessed by the research team only. Consent forms and data collection forms will be securely stored in locked cabinets at University College London. Data collection forms will not feature participants' names but a unique trial ID that could not be linked to participants by anyone outside the research team. Consent forms that identify participants will be kept separately from data collection forms.

Password-protected electronic data will be stored on the secure IT network at University College London. App usage data collected whilst using My Journey 3 will be anonymised and encrypted and will not contain personal user information.

Serious adverse events

Serious adverse events such as hospital admissions and death reported to the trial team will be reviewed by the Chief Investigator. Identified adverse events assessed as trial-related will be reported to the trial sponsor.

For peer review only

Dissemination

Results will be disseminated through scientific publications, and to a wider audience via magazines and web publications.

For peer review only

Acknowledgements: The ARIES research team are grateful to their software collaborators MyOxygen for their technical development and hosting of My Journey 3 and to Ali Mousa for his valuable contribution to the development of the original My Journey app. We are grateful to Max Birchwood for his permission to incorporate 'Back in the Saddle' in to My Journey 3. We are grateful to Rachel Perkins for her permission to adapt the Personal Recovery Plan resource and incorporate in to My Journey 3.

Author Contributions: The trial design was developed by SJ, DO, BLE and PO. SA, HR, PO and ME have led on the development of the intervention. RJ has advised on the statistical analysis. SJ is the Chief Investigator, based at University College London, DO the co-Chief Investigator, and TS the project manager. All authors have contributed and approved this manuscript.

Funding: The research is funded by the National Institute for Health Research (NIHR) Collaboration for Leadership in Applied Health Research and Care North Thames at Barts Health NHS Trust (NIHR CLAHRC North Thames). The views expressed in this article are those of the authors and not necessarily those of the NHS, the NIHR or the Department of Health and Social Care. SJ, DO and BLE are supported by the NIHR Mental Health Research Policy Unit, the NIHR Collaboration for Leadership in Applied Health Research and Care (CLAHRC) North Thames and the UCLH Biomedical Research Centre.

Data statement: The datasets generated during and/or analysed during the current study will be made available two years after the trial end.

Competing interests: None declared.

Legends:

Figure 1. SPIRIT flow diagram outlining the phases of the ARIES feasibility trial.

For peer review only

Figure 2. The My Journey 3 home screen, which is seen by the user when accessing the app on their Smartphone.

For peer review only

REFERENCES

[1] Addington J, Young J, Addington D. Social outcome in early psychosis. Psychol Med. 2003;33:1119-24.

[2] Addington J, Coldham EL, Jones B, Ko T, Addington D. The first episode of psychosis: the experience of relatives. Acta Psychiat Scand. 2003;108:285-9.

[3] The Schizophrenia Commission. The abandoned illness: a report from the Schizophrenia Commission. London: Rethink Mental Illness. 2012.

[4] Bird V, Premkumar P, Kendall T, Whittington C, Mitchell J, Kuipers E. Early intervention services, cognitive–behavioural therapy and family intervention in early psychosis: systematic review. Brit J Psychiat. 2010;197:350-6.

[5] Tsiachristas A, Thomas T, Leal J, et al. Economic impact of early intervention in psychosis services: results from a longitudinal retrospective controlled study in England. BMJ Open 2016;6:e012611. doi: 10.1136/bmjopen-2016-012611

[6] Alvarez-Jiménez M, Parker AG, Hetrick SE, McGorry PD, Gleeson JF. Preventing the second episode: a systematic review and meta-analysis of psychosocial and pharmacological trials in first-episode psychosis. Schizophrenia Bull. 2009;37:619-30.

[7] Menezes NM, Arenovich T, Zipursky RB. A systematic review of longitudinal outcome studies of first-episode psychosis. Psychol Med. 2006;36:1349-62.

[8] Mueser KT, Deavers F, Penn DL, Cassisi JE. Psychosocial treatments for schizophrenia. Annu Rev Clin Psycho. 2013;9:465-97.

[9] Mueser KT, Meyer PS, Penn DL, Clancy R, Clancy DM, Salyers MP. The Illness Management and Recovery program: rationale, development, and preliminary findings. Schizophrenia Bull. 2006;32:S32-43.

For peer review only

[10] Mueser KT, Corrigan PW, Hilton DW et al. Illness management and recovery: A review of the research. Psychiat Serv. 2002;53:1272-84.

[11] Cook JA, Copeland ME, Jonikas JA, et al. Results of a randomized controlled trial of mental illness self-management using Wellness Recovery Action Planning. Schizophrenia Bull. 2011;38:881-91.

[12] Färdig R, Lewander T, Melin L, Folke F, Fredriksson A. A randomized controlled trial of the illness management and recovery program for persons with schizophrenia. Psychiat Serv. 2011;62:606-12.

[13] Birchwood M, Spencer E, McGovern D. Schizophrenia: early warning signs. Advances in Psychiatric Treatment. 2000;6:93-101.

[14] Slade M. Personal Recovery and Mental Illness: A Guide for Mental Health Professionals. Cambridge: Cambridge University Press; 2009. (Values-Based Practice).

[15] Department of Health. Closing the Gap: Priorities for Essential Change in Mental Health. 2014, https://www.gov.uk/government/uploads/system/uploads/attachment_data/file/281250/Closing_the_gap_V2_-_17_Feb_2014.pdf (accessed August 2018)

[16] Ofcom. The Communications Market Report. London, UK: Ofcom; 2018

[17] Proudfoot J. The future is in our hands: the role of mobile phones in the prevention and management of mental disorders. Aust NZ J Psychiat. 2013;47:111-3.

[18] Firth J, Cotter J, Torous J, Bucci S, Firth JA, Yung AR. Mobile phone ownership and endorsement of "mHealth" among people with psychosis: a meta-analysis of cross-sectional studies. Schizophrenia Bull. 2015;42:448-55.

For peer review only

[19] Robotham D, Satkunanathan S, Doughty L, Wykes T. Do We Still Have a Digital Divide in Mental Health? A Five-Year Survey Follow-up. J Med Internet Res. 2016;18:e309.

[20] Naslund JA, Aschbrenner KA, Bartels SJ. How people with serious mental illness use smartphones, mobile apps, and social media. Psychiatr Rehabil J. 2016;39:364-367.

[21] Ben-Zeev D, Kaiser SM, Brenner CJ, Begale M, Duffecy J, Mohr DC. Development and usability testing of FOCUS: A smartphone system for self-management of schizophrenia. Psychiatr Rehabil J. 2013;36:289-296.

[22] Aref-Adib G, O'Hanlon P, Fullarton K, et al. A qualitative study of online mental health information seeking behaviour by those with psychosis. BMC Psychiatry. 2016;16:232.

[23] Bonet L, Izquierdo C, Escartí MJ, et al. Use of mobile technologies in patients with psychosis: A systematic review. Rev Psiquiatr Salud Ment. 2017;10:168-78.

[24] Whitehead L, Seaton P. The effectiveness of self-management mobile phone and tablet apps in long-term condition management: a systematic review. J Med Internet Res. 2016;18:e97.

[25] O'Hanlon P, Aref-Adib G, Fonseca A, et al. Tomorrow's world: current developments in the therapeutic use of technology for psychosis. BJPsych Advances. 2016;22:301-10.

[26] Bucci S, Barrowclough C, Ainsworth J, et al. Actissist: Proof-of-Concept Trial of a Theory-Driven Digital Intervention for Psychosis. Schizophrenia Bull. 2018; sby032.

[27] Chan A-W, Tetzlaff JM, Gøtzsche PC, et al. SPIRIT 2013 explanation and elaboration: guidance for protocols of clinical trials. BMJ. 2013;346:e7586

[28] National Institute for Health and Care Excellence. Implementing the Early Intervention in Psychosis Access and Waiting Time Standard: Guidance. London: NHS England Publications. 2016.

For peer review only

[29] Julious, SA. Sample size of 12 per group rule of thumb for a pilot study. Pharm Stat. 2005;287-91.

[30] Ofcom. The Communications Market Report. London, UK: Ofcom; 2017

[31] Plaistow J, Birchwood M. Back in the saddle: a guide to relapse prevention. In: Birchwood M, Fowler D, Jackson C, editors. Early Intervention in Psychosis: A Guide to Concepts, Evidence and Interventions. Chichester: McGraw-Hill; 1996. p. 239-244.

[32] Perkins R, Rinaldi M. Taking back control: a guide to planning your own recovery. South West London: St George's Mental Health NHS Trust, 2007.

[33] Torous J, Nicholas J, Larsen ME, Firth J, Christensen H. Clinical review of user engagement with mental health smartphone apps: evidence, theory and improvements. Evid Based Mental Health. 2018;21:116-9.

[34] Johnson S, Lamb D, Marston L, et al. Peer-supported self-management for people discharged from a mental health crisis team: a randomised controlled trial. Lancet. 2018;392:409-18.

[35] Tait L, Birchwood M, Trower P. A new scale (SES) to measure engagement with community mental health services. J Ment Health. 2002;11:191-8.

[36] Kay SR, Fiszbein A, Opfer LA. The positive and negative syndrome scale (PANSS) for schizophrenia. Schizophrenia Bull. 1987;13:261-76.

[37] Priebe S, Watzke S, Hansson L, et al. Objective social outcomes index (SIX): a method to summarise objective indicators of social outcomes in mental health care. Acta Psychiat Scand. 2008;118:57-63.

For peer review only

[38] Carpinello SE, Knight EL, Markowitz FE, et al. The development of the Mental Health Confidence Scale: A measure of self-efficacy in individuals diagnosed with mental disorders. Psychiatr Rehabil J. 2000;23:236-243.

[39] Neil ST, Kilbride M, Pitt L, et al. The questionnaire about the process of recovery (QPR): a measurement tool developed in collaboration with service users. Psychosis. 2009;1:145-55.

[40] Tennant R, Hiller L, Fishwick R, et al. The Warwick-Edinburgh mental well-being scale (WEMWBS): development and UK validation. Health Qual Life Out. 2007;5:63.

[41] Priebe S, McCabe RO, Bullenkamp J, et al. Structured patient–clinician communication and 1-year outcome in community mental healthcare. Brit J Psychiat. 2007;191:420-6.

[42] Sekhon M, Cartwright M, Francis JJ. Acceptability of healthcare interventions: an overview of reviews and development of a theoretical framework. BMC Health Serv Res. 2017:17:88.

For peer review only

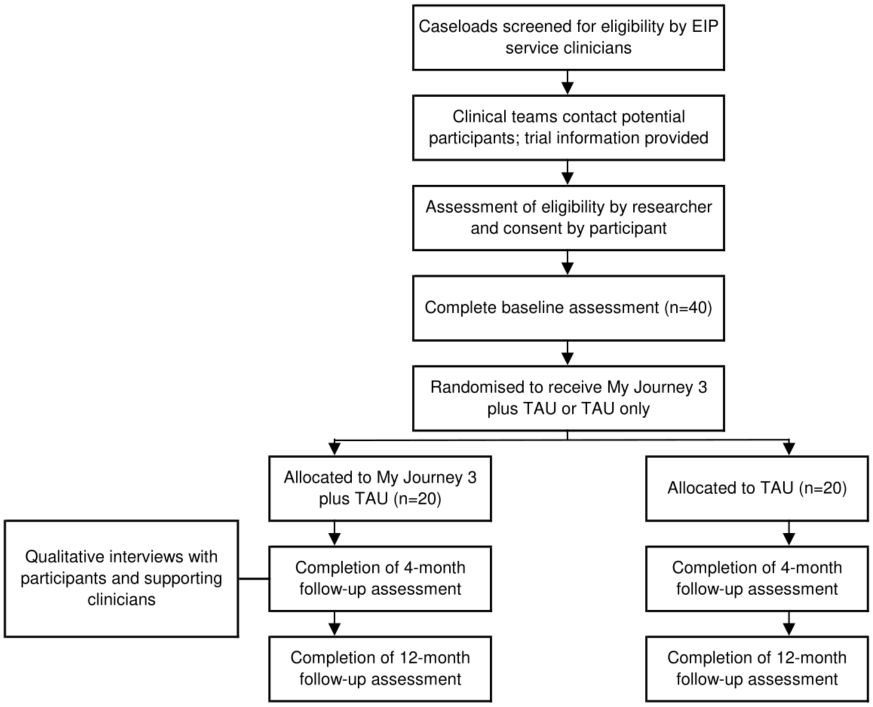

SPIRIT flow diagram outlining the phases of the ARIES feasibility trial.

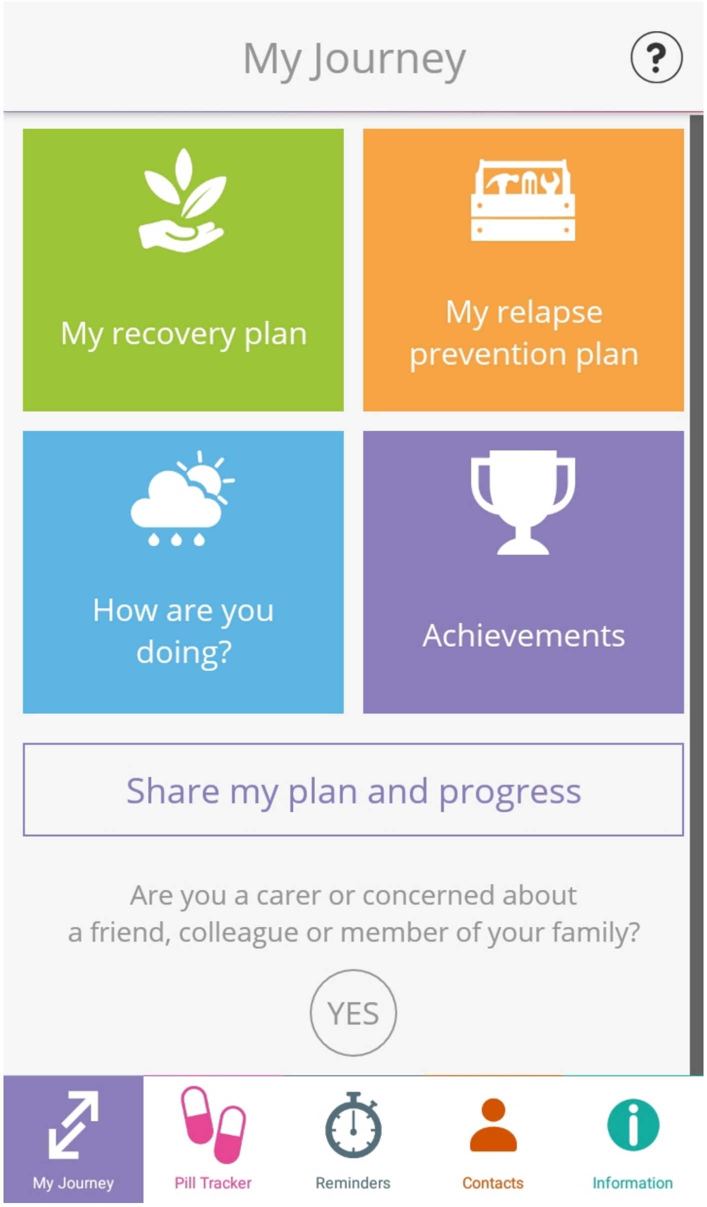

The My Journey 3 home screen, which is seen by the user when accessing the app on their Smartphone.

STANDARD PROTOCOL ITEMS: RECOMMENDATIONS FOR INTERVENTIONAL TRIALS

SPIRIT 2013 Checklist: Recommended items to address in a clinical trial protocol and related documents*

Section/item	Item No	Description	Addressed on page number
**Administrative information**			*Sections headings given until page numbers finalised*
Title	1	Descriptive title identifying the study design, population, interventions, and, if applicable, trial acronym	Title page
Trial registration	2a	Trial identifier and registry name. If not yet registered, name of intended registry	Abstract page
	2b	All items from the World Health Organization Trial Registration Data Set	Additional file 2
Protocol version	3	Date and version identifier	Ethics and dissemination - Ethical approval
Funding	4	Sources and types of financial, material, and other support	Declarations - funding
Roles and responsibilities	5a	Names, affiliations, and roles of protocol contributors	Title page/ Declarations - authors' contributions
	5b	Name and contact information for the trial sponsor	Title page

For peer review only - http://bmjopen.bmj.com/site/about/guidelines.xhtml

	5c	Role of study sponsor and funders, if any, in study design; collection, management, analysis, and interpretation of data; writing of the report; and the decision to submit the report for publication, including whether they will have ultimate authority over any of these activities	Declarations - funding
	5d	Composition, roles, and responsibilities of the coordinating centre, steering committee, endpoint adjudication committee, data management team, and other individuals or groups overseeing the trial, if applicable (see Item 21a for data monitoring committee)	Methods and analysis – Patient and Public Involvement/data monitoring and management
**Introduction**			
Background and rationale	6a	Description of research question and justification for undertaking the trial, including summary of relevant studies (published and unpublished) examining benefits and harms for each intervention	Introduction
	6b	Explanation for choice of comparators	Methods and analysis - the intervention
Objectives	7	Specific objectives or hypotheses	Introduction - aims and objectives
Trial design	8	Description of trial design including type of trial (eg, parallel group, crossover, factorial, single group), allocation ratio, and framework (eg, superiority, equivalence, noninferiority, exploratory)	Methods and analysis - design
**Methods: Participants, interventions, and outcomes**			
Study setting	9	Description of study settings (eg, community clinic, academic hospital) and list of countries where data will be collected. Reference to where list of study sites can be obtained	Methods and analysis - setting
Eligibility criteria	10	Inclusion and exclusion criteria for participants. If applicable, eligibility criteria for study centres and individuals who will perform the interventions (eg, surgeons, psychotherapists)	Methods and analysis - eligibility criteria

For peer review only

Interventions	11a	Interventions for each group with sufficient detail to allow replication, including how and when they will be administered	Methods and analysis - the intervention/ treatment as usual
	11b	Criteria for discontinuing or modifying allocated interventions for a given trial participant (eg, drug dose change in response to harms, participant request, or improving/worsening disease)	Ethics and dissemination - serious adverse events
	11c	Strategies to improve adherence to intervention protocols, and any procedures for monitoring adherence (eg, drug tablet return, laboratory tests)	Methods and analysis - the intervention
	11d	Relevant concomitant care and interventions that are permitted or prohibited during the trial	Methods and analysis - treatment as usual
Outcomes	12	Primary, secondary, and other outcomes, including the specific measurement variable (eg, systolic blood pressure), analysis metric (eg, change from baseline, final value, time to event), method of aggregation (eg, median, proportion), and time point for each outcome. Explanation of the clinical relevance of chosen efficacy and harm outcomes is strongly recommended	Methods and analysis - measures
Participant timeline	13	Time schedule of enrolment, interventions (including any run-ins and washouts), assessments, and visits for participants. A schematic diagram is highly recommended (see Figure)	Figure 1
Sample size	14	Estimated number of participants needed to achieve study objectives and how it was determined, including clinical and statistical assumptions supporting any sample size calculations	Methods and analysis - participants
Recruitment	15	Strategies for achieving adequate participant enrolment to reach target sample size	Methods and analysis - recruitment
**Methods: Assignment of interventions (for controlled trials)**			

Allocation:			
Sequence generation	16a	Method of generating the allocation sequence (eg, computer-generated random numbers), and list of any factors for stratification. To reduce predictability of a random sequence, details of any planned restriction (eg, blocking) should be provided in a separate document that is unavailable to those who enrol participants or assign interventions	Methods and analysis - randomisation
Allocation concealment mechanism	16b	Mechanism of implementing the allocation sequence (eg, central telephone; sequentially numbered, opaque, sealed envelopes), describing any steps to conceal the sequence until interventions are assigned	Methods and analysis - randomisation
Implementation	16c	Who will generate the allocation sequence, who will enrol participants, and who will assign participants to interventions	Methods and analysis - randomisation
Blinding (masking)	17a	Who will be blinded after assignment to interventions (eg, trial participants, care providers, outcome assessors, data analysts), and how	Methods and analysis - randomisation
	17b	If blinded, circumstances under which unblinding is permissible, and procedure for revealing a participant's allocated intervention during the trial	N/A
**Methods: Data collection, management, and analysis**			
Data collection methods	18a	Plans for assessment and collection of outcome, baseline, and other trial data, including any related processes to promote data quality (eg, duplicate measurements, training of assessors) and a description of study instruments (eg, questionnaires, laboratory tests) along with their reliability and validity, if known. Reference to where data collection forms can be found, if not in the protocol	Methods and analysis - data collection
	18b	Plans to promote participant retention and complete follow-up, including list of any outcome data to be collected for participants who discontinue or deviate from intervention protocols	Methods and analysis - recruitment

For peer review only - http://bmjopen.bmj.com/site/about/guidelines.xhtml

Data management	19	Plans for data entry, coding, security, and storage, including any related processes to promote data quality (eg, double data entry; range checks for data values). Reference to where details of data management procedures can be found, if not in the protocol	Methods and analysis - data monitoring and management
Statistical methods	20a	Statistical methods for analysing primary and secondary outcomes. Reference to where other details of the statistical analysis plan can be found, if not in the protocol	Methods and analysis - data analysis
	20b	Methods for any additional analyses (eg, subgroup and adjusted analyses)	N/A
	20c	Definition of analysis population relating to protocol non-adherence (eg, as randomised analysis), and any statistical methods to handle missing data (eg, multiple imputation)	Methods and analysis - data analysis
**Methods: Monitoring**			
Data monitoring	21a	Composition of data monitoring committee (DMC); summary of its role and reporting structure; statement of whether it is independent from the sponsor and competing interests; and reference to where further details about its charter can be found, if not in the protocol. Alternatively, an explanation of why a DMC is not needed	Methods and analysis - data monitoring and management
	21b	Description of any interim analyses and stopping guidelines, including who will have access to these interim results and make the final decision to terminate the trial	Methods and analysis - data analysis
Harms	22	Plans for collecting, assessing, reporting, and managing solicited and spontaneously reported adverse events and other unintended effects of trial interventions or trial conduct	Ethics and dissemination - serious adverse events
Auditing	23	Frequency and procedures for auditing trial conduct, if any, and whether the process will be independent from investigators and the sponsor	N/A
**Ethics and dissemination**			

Research ethics approval	24	Plans for seeking research ethics committee/institutional review board (REC/IRB) approval	Ethics and dissemination - ethical approval
Protocol amendments	25	Plans for communicating important protocol modifications (eg, changes to eligibility criteria, outcomes, analyses) to relevant parties (eg, investigators, REC/IRBs, trial participants, trial registries, journals, regulators)	Ethics and dissemination - ethical approval
Consent or assent	26a	Who will obtain informed consent or assent from potential trial participants or authorised surrogates, and how (see Item 32)	Methods and analysis - recruitment
	26b	Additional consent provisions for collection and use of participant data and biological specimens in ancillary studies, if applicable	N/A
Confidentiality	27	How personal information about potential and enrolled participants will be collected, shared, and maintained in order to protect confidentiality before, during, and after the trial	Ethics and dissemination - confidentiality
Declaration of interests	28	Financial and other competing interests for principal investigators for the overall trial and each study site	Declarations - competing interests
Access to data	29	Statement of who will have access to the final trial dataset, and disclosure of contractual agreements that limit such access for investigators	Methods and analysis - data monitoring and management
Ancillary and post-trial care	30	Provisions, if any, for ancillary and post-trial care, and for compensation to those who suffer harm from trial participation	N/A

For peer review

For peer review only - http://bmjopen.bmj.com/site/about/guidelines.xhtml

Dissemination policy	31a	Plans for investigators and sponsor to communicate trial results to participants, healthcare professionals, the public, and other relevant groups (eg, via publication, reporting in results databases, or other data sharing arrangements), including any publication restrictions	Ethics and dissemination – dissemination/ Methods and analysis – patient and public involvement
	31b	Authorship eligibility guidelines and any intended use of professional writers	Declarations – authors' contributions
	31c	Plans, if any, for granting public access to the full protocol, participant-level dataset, and statistical code	Data statement
**Appendices**			
Informed consent materials	32	Model consent form and other related documentation given to participants and authorised surrogates	Additional file 3
Biological specimens	33	Plans for collection, laboratory evaluation, and storage of biological specimens for genetic or molecular analysis in the current trial and for future use in ancillary studies, if applicable	N/A

*It is strongly recommended that this checklist be read in conjunction with the SPIRIT 2013 Explanation & Elaboration for important clarification on the items. Amendments to the protocol should be tracked and dated. The SPIRIT checklist is copyrighted by the SPIRIT Group under the Creative Commons "Attribution-NonCommercial-NoDerivs 3.0 Unported" license.

For peer review only - http://bmjopen.bmj.com/site/about/guidelines.xhtml

World Health Organization Trial Registration Data Set

Primary Registry and Trial Identifying Number	ISRCTN10004994
Date of Registration in Primary Registry	25/05/2018
Secondary Identifying Numbers	IRAS ID: 182553<br>REC reference: 15/LO/1453
Source(s) of Monetary or Material Support	The research is funded by the National Institute for Health Research (NIHR) Collaboration for Leadership in Applied Health Research and Care North Thames at Barts Health NHS Trust (NIHR CLAHRC North Thames).
Primary Sponsor	Camden and Islington NHS Foundation Trust
Secondary Sponsor(s)	N/A
Contact for Public Queries	Thomas Steare<br><br>+44 (0)20 7679 8192<br>thomas.steare.15@ucl.ac.uk<br>UCL Division of Psychiatry<br>6th Floor, Maple House<br>149 Tottenham Court Road<br>London<br>W1T 7NF
Contact for Scientific Queries	Prof Sonia Johnson<br><br>+44 (0)20 7679 9453<br>s.johnson@ucl.ac.uk<br>UCL Division of Psychiatry<br>6th Floor, Maple House<br>149 Tottenham Court Road<br>London<br>W1T 7NF
Public Title	ARIES: App to support Recovery in Early Intervention Services
Scientific Title	App to support Recovery In Early intervention Services (the ARIES study): feasibility trial of a supported self-management Smartphone application for psychosis
Countries of Recruitment	England
Health Condition(s) or Problem(s) Studied	First-episode psychosis
Intervention(s)	Self-management Smartphone app (My Journey 3)
Key Inclusion and Exclusion Criteria	Participant inclusion criteria

For peer review only

	1. Currently on the caseload of an Early Intervention Service and in contact with clinicians<br>2. Aged 16 or older<br>3. Have a diagnosis of psychosis<br>4. Own an Android Smartphone.<br><br>Participant exclusion criteria<br><br>1. Lack capacity to provide consent to take part in the study<br>2. Unable to communicate and understand English sufficiently to understand trial procedures and use the app<br>3. In the view of their EIS team, pose such a high risk to others that it would be unsafe to conduct research meetings even on NHS premises.
Study Type	Multi-centre randomised controlled feasibility trial
Date of First Enrolment	09/03/2017
Sample Size	40
Recruitment Status	Recruitment target met – no longer recruiting
Primary Outcome(s)	Relapse as indicated by admission to acute care (inpatient wards, crisis resolution teams, crisis houses and acute day services) during the 12-month follow-up period. Data on admissions to acute care during the trial period is collected from patient records at the 12-month follow-up.
Key Secondary Outcomes	1. Social outcomes are measured using The Social Outcomes Index (Priebe, Watzke, Hansson & Burns, 2008) at the study baseline meeting, at a 4-month follow-up meeting and at a 12-month follow-up meeting<br>2. Mental wellbeing is assessed using The Mental Health Confidence Scale (Carpinello et al., 2000) and The Warwick-Edinburgh Mental Well-Being Scale (NHS Health Scotland, University of Warwick & University of Edinburgh, 2007) at the study baseline meeting, at a 4-month follow-up meeting and at a 12-month follow-up meeting<br>3. Recovery in psychosis is assessed using The Process of Recovery Questionnaire (Neil et al., 2009) at the study baseline meeting, at a 4-month follow-up meeting and at a 12-month follow-up meeting<br>4. Quality of life and satisfaction with treatment is assessed using The DIALOG scale (Priebe et al., 2007) at the study baseline meeting, at a 4-month follow-up meeting and at a 12-month follow-up meeting<br>5. Positive, negative and general psychopathology symptoms are assessed using the PANSS (Kat et al., 1987) at the study

For peer review only

	baseline meeting, at a 4-month follow-up meeting and at a 12-month follow-up meeting<br>6. Participants' engagement with Early Intervention Services during the study period is obtained using the Service Engagement Scale (SES; Tait et al., 2002) completed by participants' clinicians at baseline and at the 12-month follow-up.<br>7. The following patient information is collected from patient records at baseline and one year after entry into the study:<br>7.1. Current diagnosis<br>7.2. Current care cluster<br>7.3. Care plan approach status<br>8. The usability and acceptability of My Journey 3 for service users and clinicians is assessed from semi-structured qualitative interviews conducted at the 4-month follow-up meeting.
Ethics Review	National Research Ethics Service Committee London - Brent, 02/10/2015, ref: 15/LO/1453. Amendment approved 29/07/2017.
Completion date	Study is ongoing

For peer review only

Service user participant consent form

Study Title: App to support Recovery In Early intervention Services (the ARIES study): Pilot randomised controlled trial of a self-management smartphone application

Principal Investigators: Professor Sonia Johnson and Professor David Osborn

Please Initial Each Box

1. I confirm that I have read and understood the Participant Information Sheet V5 dated 29/05/2017 for the above study and have had the opportunity to ask questions about the study. ☐
2. I understand that my participation is voluntary and that I am free to withhold personal information or to withdraw my participation at any time, without giving any reason, and without my medical care or legal rights being affected. ☐
3. I understand that if I choose to withdraw from the study that any data that I have already provided for the purposes of the research will be kept and used by the research team. ☐
4. I give permission for my General Practitioner (GP) and my Early Intervention team to be told I am participating in this study. ☐
5. I understand that relevant sections of my medical notes and data collected during the study may be looked at by regulatory authorities or from the NHS Trust, where it is relevant to my taking part in this research. I give permission for these individuals to have access to my records. ☐
6. I understand that I will be given a £20 gift as cash for my participation in each study assessment. ☐
7. I agree to the research team consulting NHS electronic records to investigate my diagnosis, medication, and mental health service use, and give them permission to do so even if I choose to no longer participate in the intervention, or they are not able to carry out further study interviews with me. ☐
8. I understand that in the event that I disclose information which may indicate new risk to myself or others, the researcher will be obliged to follow NHS Trust risk procedures that may require release of my personal data. ☐
9. I give permission for findings from the study to be written up for publication. Any publication will not identify me. ☐
10. I give permission to be audio recorded where required for the purposes of the study. I understand these audio-recordings will be transcribed and anonymised and audio recordings destroyed after the study. I give permission for direct quotations taken from this interview to be included in papers written for publication. Any quotation would not identify me. ☐
11. I give permission for the research team to collect data from the My Journey 3 app regarding the frequency, duration, and pattern of my use of it. I understand that no personal information will be collected from the app. ☐
12. I give permission for non-identifiable data to be shared with other research teams for research purposes. ☐

For peer review only

For peer review only - http://bmjopen.bmj.com/site/about/guidelines.xhtml

13. I agree to take part in this study. ☐

Name of participant	Date	Signature
Name of Researcher taking consent	Date	Signature

For peer review only

For peer review only - http://bmjopen.bmj.com/site/about/guidelines.xhtml
